# Geographic and longitudinal variations of anatomical characteristics and mechanical properties in three bamboo species naturally grown in Lombok Island, Indonesia

**DOI:** 10.1038/s41598-023-29288-3

**Published:** 2023-02-08

**Authors:** Dwi Sukma Rini, Futoshi Ishiguri, Ikumi Nezu, Agus Ngadianto, Denny Irawati, Naoki Otani, Jyunichi Ohshima, Shinso Yokota

**Affiliations:** 1 School of Agriculture, Utsunomiya University, Utsunomiya, 321-8505 Japan; 2 United Graduate School of Agricultural Science, Tokyo University of Agriculture and Technology, Fuchu, Tokyo 183-8509 Japan; 3 Department of Forestry, University of Mataram, Mataram, 83125 Indonesia; 4 Vocational College, Universitas Gadjah Mada, Yogyakarta, 55281 Indonesia; 5 Faculty of Forestry, Universitas Gadjah Mada, Yogyakarta, 55281 Indonesia; 6 Tochigi Prefectural Forestry Research Center, Utsunomiya, 321-2105 Japan

**Keywords:** Forestry, Mechanical properties

## Abstract

The anatomical characteristics (fiber length and fiber area) and mechanical properties (modulus of elasticity, modulus of rupture, compressive strength, tensile Young’s modulus, and tensile strength) of *Bambusa vulgaris*, *Bambusa maculata*, and *Gigantochloa atter*, naturally growing at four different sites in Lombok Island, Indonesia, were examined for evaluating geographic and longitudinal variations by mixed-effects modeling to effectively utilize bamboo culm resources for structural materials. We found geographic and longitudinal variations of bamboo culm properties in these three species. Based on the results, we concluded that, for utilization of bamboo culm as a structural material, variation of individual culm rather than site, and longitudinal variations should be considered for *Bambusa* species and *G. atter*, respectively.

## Introduction

Bamboo has been regarding as a fast-growing sustainable building material with a simple manufacturing process^1,2^. Thus, it is expected to be an alternative material instead of conventional building materials, such as concrete, steel, and timber^2^. The bamboo culm wall has a distinctive structure: the culm is composed of parenchymatous ground tissue with embedded vascular bundles^3^. The vascular bundles are composed of metaxylem vessels and sclerenchyma fiber sheaths that serve as transportation function and mechanical support^3,4^.

Understanding the variations in bamboo culm properties including anatomical characteristics is necessary to utilize bamboo as a building material. Bamboo culm properties vary between and within species in some bamboo species, such as *Bambusa vulgaris*, *B. blumeana*, *B. balcoa*, *B. rigida*, *Dendrocalamus strictus*, *D. asper*, *Gigantochloa scortechinii*, *G. atter*, *G. pruriens*, *Phyllostachys pubescens*, *P. edulis*, and *Schizostachyum* species^5–16^. For example, longitudinal variations of culm properties were found to be within-species variations^6,9,15^. The modulus of elasticity (MOE) and modulus of rupture (MOR) tended to increase from the base to the top of the culm in several species of the genera *Gigantochloa* ^9^, but the MOE in *G. scortechinii* increased from the base to the middle of the height and then stabilized towards the top of the culm^9,15^. On the other hand, MOR of *G. scortechinii* was relatively stable along the longitudinal direction^15^. In genera *Bambusa*, the physical and mechanical properties tended to increase from the base to the top of the culm^8,9,11^, whereas fiber length varied in several species^6,9,11^. These results suggested that longitudinal variation patterns of culm properties might differ among species and culm properties. Thus, the longitudinal variation patterns should be clarified for different bamboo species and different culm properties to effectively utilize these culms.

In addition, culm properties have geographic variations^17–19^. Yang et al.^17^ examined fiber dimension and chemical composition of 3-year-old *Bambusa chungii* culms collected from eight provenances, China. They found significant among provenance variations in fiber length^17^. Similar among provenance differences in fiber length were found in *Fargesia yunnanensis* in China^18^ and *Dendrocalamus giganteus*^19^ collected in Yunnan, China. Variations within-species and among sites should be clarified to effectively utilize bamboo culm as modern construction materials.

*B. vulgaris*, *B. maculata*, and *G. atter* are common bamboo species in Indonesia^20^. These three bamboo species grow naturally on Lombok Island, Indonesia^21,22^. Based on traditional knowledge, local communities have been using them as fences, bridges, scaffolding, furniture, room partitions, and traditional houses. Unfortunately, detailed scientific information is still limited on the culm properties of these three bamboo species for utilizing their culms as modern construction materials. Previously, we examined the longitudinal and geographic variation of the green moisture content and basic density of the three bamboo species by application of mixed-effects modeling^23^. From the bottom to the top of the culm, the green moisture content decreased, and the basic density increased in all species. In addition, we found that the main factor affecting the longitudinal variation in both properties was individual culm variance rather than site variance. Furthermore, a large geographic variation of these properties was found in *G. atter*. Unfortunately, other culm properties such as anatomical characteristics and mechanical properties were not investigated yet for the three species. Therefore, detailed information about other properties is required for the effective and sustainable utilization of the three bamboo species. The objectives of this study were to determine the longitudinal and geographic variations in the anatomical characteristics (fiber length and fiber area) and mechanical properties (MOE, MOR, compressive strength parallel to the grain [CS], tensile Young’s modulus parallel to the grain [TM], and tensile strength parallel to the grain [TS]) of *B. vulgaris*, *B. maculata*, and *G. atter*, naturally grown in Lombok Island, Indonesia for utilizing these culm as modern construction materials.

## Materials and methods

### Sampling sites and sample preparation

**Figure 1:**
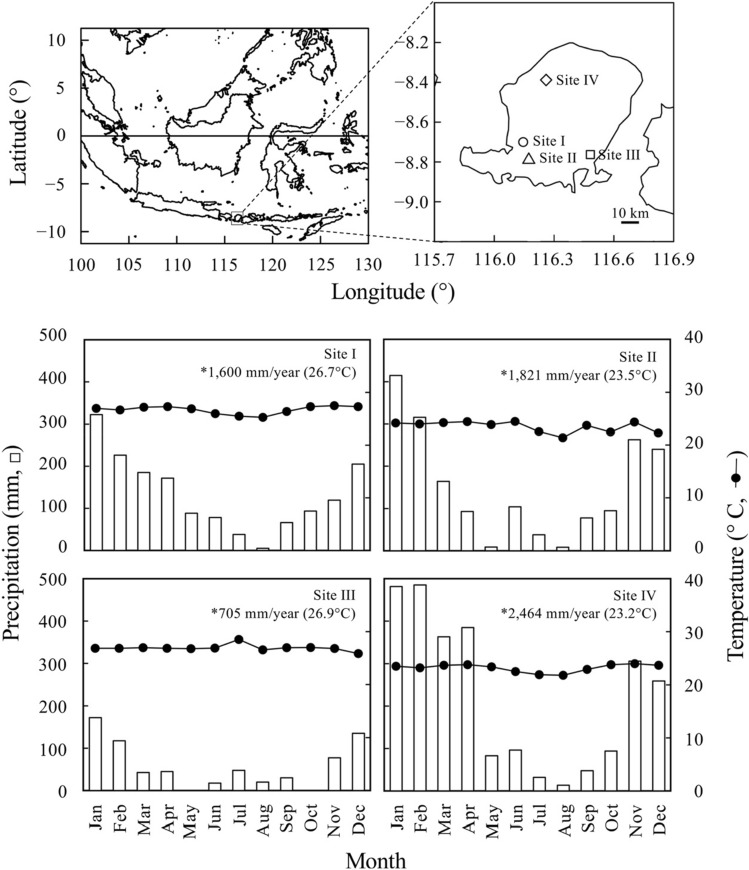
Locations and climate conditions of sampling sites in the present study^23^. Note: Site I, Tempos (8°41′59" S, 116°8′40" E); Site II, Kabul (8°47′21" S, 116°10′21" E); Site III, Keruak (8°45′45" S, 116°28′54" E); Site IV, Genggelang (8°23′16" S, 116°15′35" E). *, mean annual precipitation. The value in the bracket is the mean annual temperature. Climate data were provided from Nusa Tenggara River Basin Management I, Indonesia. Mean monthly temperature and precipitation were calculated by averaging monthly values from 2016 to 2018. Bars indicate the mean values of precipitation. Circles indicate the mean values of temperature. The graph was originally created by R^27^ (version 4.0.3, https://www.R-project.org/).

**Figure 2:**
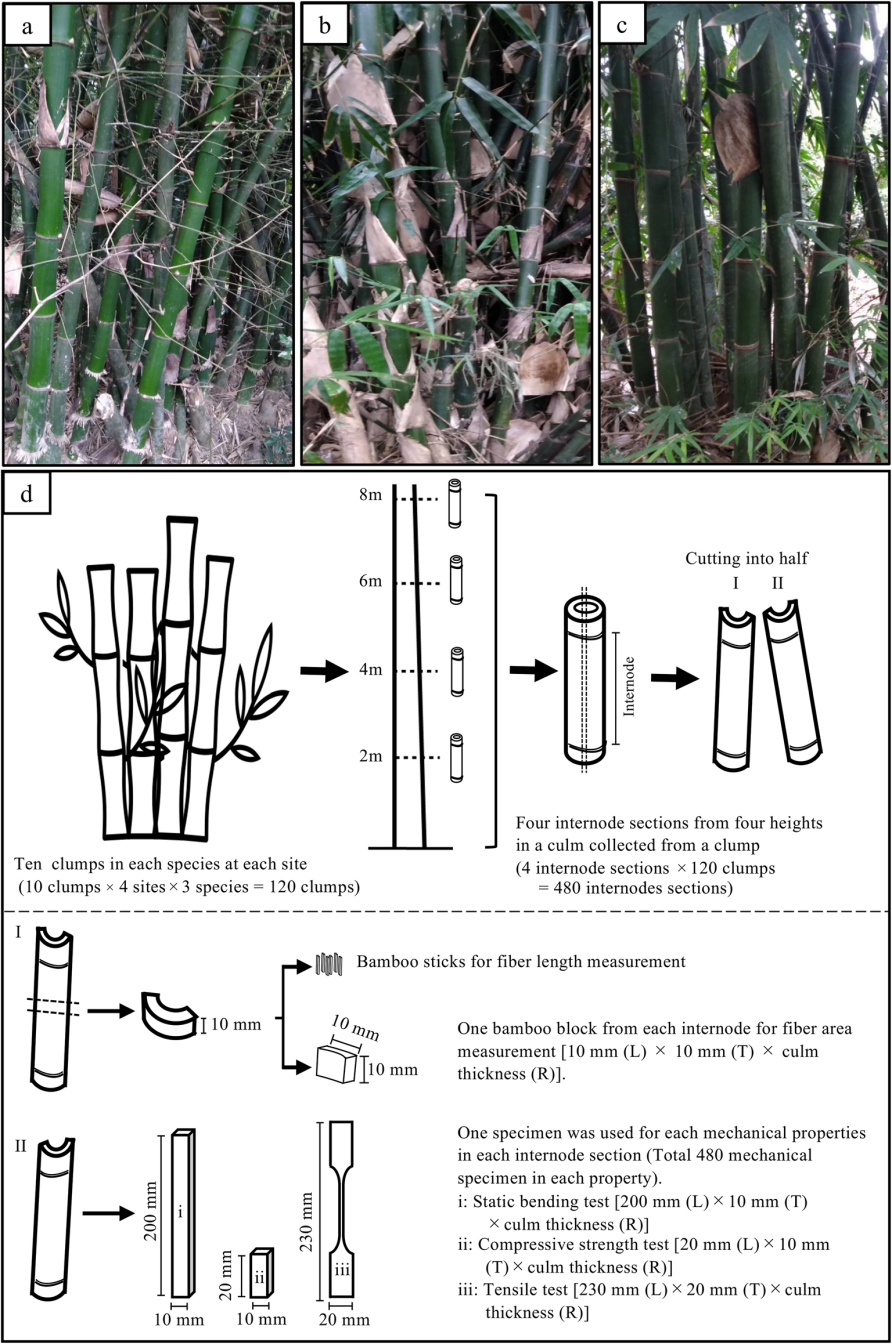
Photographs of the clumps in three bamboo species (**a**–**c**) and schematic diagrams of experimental procedures (**d**). Note: a, *B. vulgaris*; b, *B. maculata*; c, *G. atter*. The specimens of fiber area measurement and mechanical properties have the whole culm thickness (including the cortex and inner part of the culm) in the radial direction.

Culms of three- to four-year-old of *Bambusa vulgaris* Schrad. ex J.C., *B. maculata* Widjaja, and *Gigantochloa atter* (Hassk) Kurz ex Munro were collected from naturally bamboo forests at four sites in Lombok Island, Indonesia^23^. The culm age was estimated based on some morphological features (the presence of culm sheath, color, and sound created by tapping with fingers) checked by an experienced bamboo farmer. Figure 1 shows the map of sampling sites and climatic conditions of the sites. Ten individual culms in each species at each site were collected from different clumps and cut 20 cm above the ground (Fig. 2). A total of 120 culms (three species × four sites × 10 individual culms from 10 individual clumps) were collected in the present study (Fig. 2). To determine the longitudinal variations of the anatomical characteristics and mechanical properties, the internode section was collected at 2-m intervals from 2 to 8 m above the ground; a total of 480 internode sections. (120 culms × four heights) were obtained from three species (Fig. 2). The collection of bamboo culms was permitted by Indonesian Institute of Science (Reference no. B-206/SKIKH/KS.02.04/X/2020) and complied with relevant guidelines and regulations of Indonesian CITES Management Authority, Ministry of Environment and Forestry, Indonesia. In addition, the voucher specimen was deposited at the Herbarium Lesser Sunda, University of Mataram, Indonesia under the voucher number of DSR01, 02, and 03 (specimens were identified by Mr. Niechi Valentino). Table 1 shows the culm diameter at 1.3 m above the ground, total culm height, and mean value of culm thickness at four positions^23^.

**Table 1:** Mean values and standard deviations of growth characteristics in three bamboo species at each site^23^.

Species	Site	*n*	CD (cm)	CH (m)	CT (cm)
*B. vulgaris*	I	10	8.0 ± 0.2	17.53 ± 1.38	0.90 ± 0.12
	II	10	8.0 ± 0.2	15.18 ± 1.90	0.66 ± 0.06
	III	10	6.8 ± 0.4	15.92 ± 1.78	0.76 ± 0.05
	IV	10	7.1 ± 0.2	13.59 ± 1.56	0.85 ± 0.12
*B. maculata*	I	10	8.0 ± 0.2	16.78 ± 1.63	0.61 ± 0.09
	II	10	7.7 ± 0.4	14.91 ± 1.71	0.74 ± 0.06
	III	10	6.3 ± 0.3	14.77 ± 1.45	0.81 ± 0.06
	IV	10	7.0 ± 0.6	16.41 ± 1.49	0.56 ± 0.05
*G. atter*	I	10	9.3 ± 0.4	18.10 ± 1.39	0.68 ± 0.06
	II	10	9.4 ± 0.3	16.42 ± 2.01	0.87 ± 0.08
	III	10	7.9 ± 0.4	14.92 ± 1.44	0.49 ± 0.04
	IV	10	8.4 ± 0.2	17.05 ± 2.03	0.77 ± 0.06

*n* number of individual culms; *CD* culm diameter at 1.3 m above the ground; *CH* culm height; *CT* mean values of culm thickness from those at four height positions. Values followed by mean value indicate standard deviations.

### Anatomical characteristics

The internode sections were split into two parts: the strips (10 mm in the longitudinal direction) and the small blocks (10 [T] mm by 10 [L] mm by culm thickness in the radial direction) (Fig. 2). The strips and small blocks were the samples for measuring fiber length and fiber area, respectively. In the present study, the fiber area was defined as the sheaths area around the vascular bundles^24^.

To determine the fiber length, small sticks (not including the cortex and the most inner part of the culm) were obtained from the strips with a razor blade (Fig. 2). Randomly selected sticks from each height position (without separation of collected positions of the samples within the radial direction of the culm in a height) were macerated with Schultze’s solution (100 mL of 35% nitric acid containing 6 g potassium chloride) at 70 °C for two hours. The length of 50 fibers was measured in each sample with a digital caliper (CD-15CX, Mitutoyo, Kawasaki, Japan) on a microprojector (V-12B, Nikon, Tokyo, Japan).

**Figure 3:**
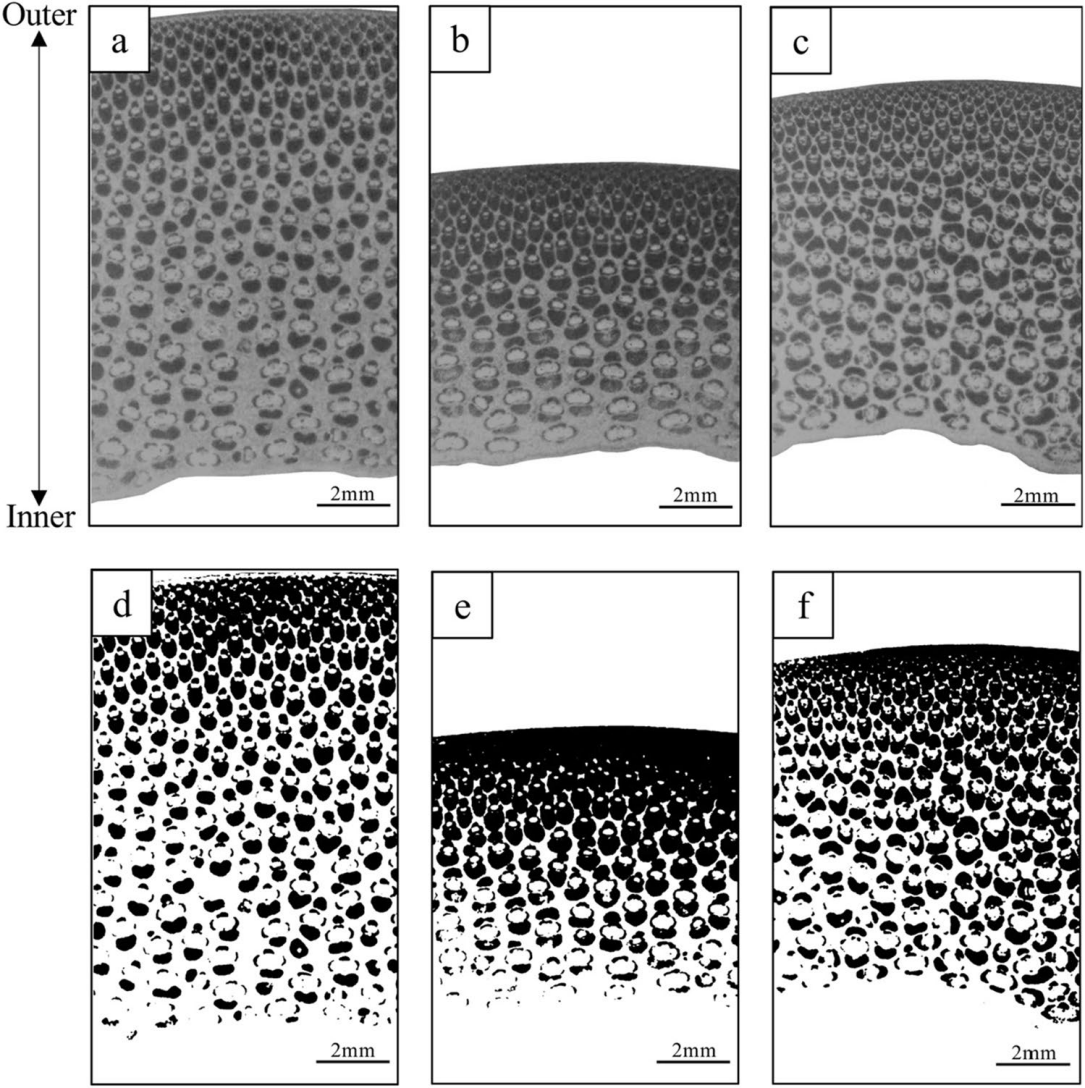
The photomicrographs of transverse section in *B. vulgaris* (**a** and **d**), *B. maculata* (**b** and **e**), and *G. atter* (**c** and **f**). Note: a, b and c, original image; d, e and f, binarized image processed by ImageJ^25^ (version 1.53e, https://imagej.nih.gov/ij/). The darker area in photomicrographs (**d**, **e** and **f**) is fiber sheath area.

To measure the fiber area, one block was taken at each height position on each individual culm (Fig. 2). The transverse sections of the blocks were polished with sandpaper sheet (#180, 3 M Japan, Tokyo, Japan), and then their images were captured using a microscope digital camera (DS-2210, Sato Shouji Inc., Kawasaki, Japan) attached to a stereo microscope (SZX12, Olympus, Tokyo, Japan). The fiber area was determined by ImageJ^25^ (version 1.53e). Binarized images were prepared by ImageJ to distinguish as clearly as possible between the vascular bundle and the background (Fig. 3). The darker area of binarized images in Fig. 3 was identified as fiber sheaths. The fiber area was calculated as follows:

1 $$FA\left( \% \right) \, = A_{fs} /A_{c} \times {1}00$$

where *FA* = fiber area (%), *A*_*fs*_ = the transverse-sectional area of fiber sheath in bamboo culm (mm^2^), and *A*_*c*_ = the transverse-sectional area of bamboo culm (mm^2^).

### Mechanical properties

The following mechanical properties of culm were measured: bending properties (MOE and MOR), CS, and tensile properties (TM and TS). A total of 480 specimens (one specimen × four heights in an individual × ten individuals × three species × four sites) without node were obtained in each property (Fig. 2).

The strips (10 [T] mm × 200 [L] mm × varied culm thickness in the radial direction) were prepared as the specimens for the static bending test (Fig. 2). The static bending test was conducted using a universal testing machine (MSC 5/500–2, Tokyo Testing Machine, Tokyo, Japan). A load was applied to the center of the specimen on the outer cortex surface with 180 mm span and 3 mm min^−1^ load speed. Due to larger thickness (exceeded 12.9 mm = 180 mm of span / 14) in the radial direction, the span / depth ratio in some specimens was less than 14, indicating that MOR in some specimens might be underestimated due to the occurrence of the shearing strength^26^. Of 480 specimens, the large culm thickness exceeded 12.9 mm was total 19 specimens from *B. vulgaris* species collected at 2 m height position from different sites (Site I = four specimens, Site II = six specimens, Site III = four specimens, and Site IV = five specimens). However, all these 19 specimens were broken at the tension side of the specimens during static bending test, which was the normal breaking forms of bending specimens with span / depth ratio less than 14.

The load and deflection were recorded with a personal computer, and then MOE and MOR were calculated by the following formulae:

2 $$MOE \, \left( {GPa} \right) \, = \Delta Pl^{3} / \, 4\Delta Ybh^{3} \, \times 10^{ - 3}$$

3 $$MOR \, \left( {MPa} \right) \, = \, 3Pl/ \, 2bh^{2}$$

where *ΔP* = difference between upper and lower proportional limit within the range of elasticity (N), *l* = length of the span (mm), *∆Y* = deflection due to *∆P* (mm), *b* = width of the specimen (mm), *h* = height of the specimen (mm), and *P* = maximum load (N).

The compressive test specimen (10 [T] mm × 20 [L] mm × culm thickness in the radial direction) was also prepared (Fig. 2). The test was conducted using a universal testing machine (RTF-2350, A&D, Tokyo, Japan) with a load speed of 0.3 mm min^−1^. The compressive strength parallel to grain (CS) was calculated by the following formula:

4 $${\text{CS }}\left( {{\text{MPa}}} \right) \, = P/A_{0}$$

where *P* = maximum load (N), and *A*_0_ = the cross-sectional area of the specimen (mm^2^).

The tensile tests were conducted using bone-shaped specimens (Fig. 2). The specimen length was 230 (L) mm with a 20 (T) mm width of the specimen grip. The cross-sectional area of the specimen was 2 mm in the tangential direction by culm thickness in the radial direction. A strain gage type extensometer (SG25-10A, A&D, Tokyo, Japan) was used to detect the elongation in the test specimen. The specimen grip sections were attached to small boards (75 mm in length × 40 mm in width × 5 mm in thickness) and then were clamped between the metal grip of a universal testing machine (RTC-2410, A&D, Tokyo, Japan). The tensile load was applied at 1 mm min^−1^. The tensile strength (TS) and Young’s modulus (TM) were calculated by the following formulae:

5 $${\text{TS }}\left( {{\text{MPa}}} \right) \, = P/A_{0}$$

6 $${\text{TM }}\left( {{\text{GPa}}} \right) \, = \Delta Pl/A_{0} \Delta l \times {1}0^{{ - 3}}$$

where *P* = maximum load (N), *A*_*0*_ = the cross-sectional area of the specimen (mm^2^), *∆P* = difference between upper and lower proportional limit within the range of elasticity (N), *l* = gauge length (mm), and *∆l* = elongation of the original gauge length (mm).

The moisture content and air-dry density of each specimen were measured after each mechanical testing by the oven-dry method. The moisture content and air-dry density of the specimen at testing were listed in Table S1.

### Statistical analysis

The statistical analyses were conducted using R software (version 4.0.3)^27^. To evaluate the longitudinal variations of the measured properties in each species, the *y*-intercept, linear, and nonlinear mixed-effects models with each measured property value as a responsible variable, the height position as a fixed effect, and site and individual culm as random effects were developed by the “lmer” function in “lme4” packages^28^ and the “nlme” function in the “nlme” package^29^. The following four full models were developed and compared:

Model I (*y*-intercept model):

7 $$Y_{ijk} = \alpha_{{1}} + Site_{{{1}k}} + Culm_{{{1}jk}} + e_{ijk}$$

Model II (linear model):

8 $$Y_{ijk} = \, (\beta_{0} + Site_{0k} + Culm_{0jk} )X_{ijk} + \beta_{{1}} + Site_{{{1}k}} + Culm_{{{1}jk}} + e_{ijk}$$

Model III (logarithmic model):

9 $$Y_{ijk} = \, (\gamma_{0} + Site_{0k} + Culm_{0jk} ){\text{ ln }}\left( {X_{ijk} } \right) + \gamma_{{1}} + Site_{{{1}k}} + Culm_{{{1}jk}} + e_{ijk}$$

**Figure 4:**
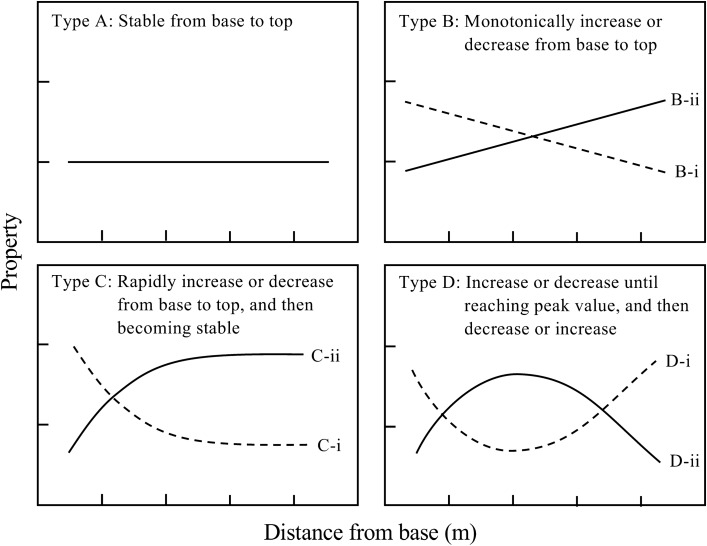
Classification of longitudinal variation of bamboo culm property. Note: Lines or curves indicate formulae with fixed-effect parameters in the selected mixed-effect model for explaining longitudinal variation (Tables 3, 4, 5). Coefficient of variation calculated from mean values and standard deviation from 2 to 8 m above the ground estimated by fixed-effect parameters values less than 3.0% is regard as stable variation (Type A), even in selected model is Model II to IV.

Model IV (quadratic model):

10 $$\begin{gathered} Y_{ijk} = \, (\zeta_{0} + Site_{0k} + Culm_{0jk} )X_{ijk}^{{2}} + \, (\zeta_{{1}} + Site_{{{1}k}} + Culm_{{{1}jk}} )X_{ijk} \hfill \\ + \zeta_{{2}} + Site_{{{2}k}} + Culm_{{{2}jk}} + e_{ijk} \hfill \\ \end{gathered}$$

where *Y*_*ijk*_ is measured property at the *i*th height position from the *j*th individual culm within the *k*th site, *X*_*ijk*_ is the *i*th height position from the *j*th individual culm within the *k*th site, *α*_1_, *β*_0_, *β*_1_, *γ*_0_, *γ*_1_, *ζ*_0_, *ζ*_1_, and *ζ*_2_ are the fixed effects, *Site*_0*k*_, *Site*_1*k*_, and *Site*_2*k*_ are the random effect at the site level, *Culm*_0*jk*_, *Culm*_1*jk*_, and *Culm*_2*jk*_ are the random effects at the individual culm level, and *e*_*ijk*_ is residual. Total 36 derived models (three *y*-intercept models, 15 linear models, nine logarithmic models, and nine quadratic models) were developed. The model selection was conducted using the Akaike information criterion^30^. The model with the minimum AIC value was regarded as the most parsimonious model among developed models. In addition, the differences in AIC (*Δ*AIC) ≤ 2 indicate no significant differences between models, and a simpler model with fewer parameters is preferred^31^. To evaluate the longitudinal variation, estimated values of each property was calculated at 0.1 m interval from 2.0 to 8.0 m above the ground using fixed-effect parameters of the selected models. Mean value and standard deviation were obtained from the estimated values from 2.0 to 8.0 m in each property. In addition, the coefficient of variation was also calculated from the mean value and standard deviation. The longitudinal variation patterns were classified into four types (Types A to D) based on the model selection (Fig. 4). Although model II to IV was selected, longitudinal variation with the coefficient of variation less than 3.0% was regarded as stable (Type A in Fig. 4).

Geographic variations in each bamboo property were estimated by evaluating the variance component of sites and culms as random effects by using the intercept-only linear mixed-effects model. The full model is described as follows:

11 $$Y_{ijk} = \mu + Site_{k} + Culm_{jk} + e_{ijk}$$

where *Y*_*ijk*_ is the bamboo property at the *i*th height position of the *j*th individual culm within *k*th site, *μ* is the model intercept or grand mean, *Site*_*k*_ is the random effect of the *k*th site, *Culm*_*jk*_ is random effect of *j*th individual culm within *k*th site, and *e*_*ijk*_ is the residual. The contribution of each level of variation was calculated as a percentage of the total random variation in the best model^32,33^.

## Results

The statistical values of the anatomical characteristics and mechanical properties of the three bamboo species are summarized in Table 2. For the fiber length, *G. atter* showed the longest value, whereas the shortest value was found in *B. maculata*. Among the three species, *B. maculata* had the highest mean fiber area (Table 2 and Fig. 3) and *B. vulgaris* had the lowest. For mechanical properties, *B. vulgaris* showed the lowest value for MOE, CS, and TM. *Bambusa maculata* showed the highest value for MOR, CS, and TS. In addition, *G. atter* showed the highest value for MOE and TM, although they are almost similar to *B. maculata*. The lowest value for MOR and TS was found in *G. atter*.

**Table 2:** Statistical values of the fiber length, fiber area, and mechanical properties in the three bamboo species.

Species	Statistical value	FL (mm)	FA (%)	MOE (GPa)	MOR (MPa)	CS (MPa)	TM (GPa)	TS (MPa)
BV	Mean	3.19	39.3	13.16	168.1	64.7	21.02	223.0
	SD	0.14	3.4	1.76	18.0	7.6	2.52	26.7
	Minimum	2.83	32.9	10.28	125.0	50.8	15.16	164.5
	Maximum	3.73	46.3	16.90	211.5	82.4	25.18	274.9
BM	Mean	3.07	46.7	14.59	177.3	68.1	23.08	226.6
	SD	0.22	4.3	2.09	23.9	9.6	3.45	31.4
	Minimum	2.60	37.3	11.11	133.1	49.7	26.24	148.0
	Maximum	3.47	56.0	20.09	223.0	86.9	30.54	280.9
GA	Mean	3.41	45.0	15.07	161.7	65.9	23.62	206.5
	SD	0.14	6.2	2.24	23.8	11.1	4.12	30.5
	Minimum	3.05	29.4	9.26	107.6	44.0	14.27	150.9
	Maximum	3.70	60.9	19.24	214.5	84.7	33.87	272.7

The total number of individual culms in each species = 40 culm; *BV B. vulgaris*; *BM B. maculata*; *GA G. atter*; *SD* standard deviation; *FL* fiber length; *FA* fiber area; *MOE* modulus of elasticity; *MOR* modulus of rupture; *CS* compressive strength parallel to grain; *TM* tensile Young’s modulus; *TS* tensile strength.

**Table 3:** Parameter estimates, standard errors, and* p* values of fixed-effect parameters, and standard deviations of the random effect estimates for the most parsimonious models of culm properties in *B. vulgaris.*

Property	Eq	Fixed-effect parameter									Random effect						
		*α*_1_									Standard deviation						
		Estimate	SE	*p *value							*Site*_1*k*_	*Culm*_1*jk*_	*e*_*ijk*_				
FA	I	39.324	0.535	< 0.001							–	2.531	4.499				
TM	I	21.016	0.398	< 0.001							–	982	4.637				

Property	Eq	*β*_0_			*β*_1_						Standard deviation						
		Estimate	SE	*p *value	Estimate	SE	*p *value				*Site*_0*k*_	*Culm*_0*jk*_	*Site*_1*k*_	*Culm*_1*jk*_	*e*_*ijk*_		
CS	II	0.832	0.449	0.146	60.508	1.512	< 0.001				0.805	–	–	6.701	5.573		

Property	Eq	*γ*_0_			*γ*_1_						Standard deviation						
		Estimate	SE	*p *value	Estimate	SE	*p *value				*Site*_0*k*_	*Culm*_0*jk*_	*Site*_1*k*_	*Culm*_1*jk*_	*e*_*ijk*_		
FL	III	− 1.138	0.036	< 0.001	4.896	0.059	< 0.001				–	–	0.035	–	0.235		
TS	III	2.733	6.774	0.687	218.973	10.38	< 0.001				–	10.068	–	–	43.36		

Property	Eq	*ζ*_0_			*ζ*_1_			*ζ*_2_			Standard deviation						
		Estimate	SE	*p *value	Estimate	SE	*p *value	Estimate	SE	*p value*	*Site*_0*k*_	*Culm*_0*jk*_	*Site*_1*k*_	*Culm*_1*jk*_	*Site*_2*k*_	*Culm*_2*jk*_	*e*_*ijk*_
MOE	IV	− 0.200	0.025	< 0.001	2.525	0.257	< 0.001	6.587	0.714	< 0.001	–	–	–	–	0.753	1.417	1.281
MOR	IV	− 1.355	0.254	< 0.001	16.777	2.578	< 0.001	125.108	6.233	< 0.001	–	–	–	–	–	16.636	12.838

*FL* fiber length; *FA* fiber area; *MOE* modulus of elasticity; *MOR* modulus of rupture; *CS* compressive strength parallel to grain; *TM* tensile Young’s modulus; *TS* tensile strength; Eq., fitted model; *SE* standard error; *α*_1_, *β*_0_, *β*_1_, *γ*_0_, *γ*_1_, *ζ*_0_, *ζ*_1_, and *ζ*_2_, the fixed effects; *Site*_0*k*_, *Site*_1*k*_, and *Site*_2*k*_*,* the random effect at the site level; *Culm*_0*jk*_, *Culm*_1*jk*_, and *Culm*_2*jk*_ the random effect at the individual culm level; *e*_*ijk*_, residual.

**Table 4:** Parameter estimates, standard errors, and *p* values of fixed-effect parameters, and standard deviations of the random effect estimates for the most parsimonious models of culm properties in *B. maculata.*

Property	Eq	Fixed-effect parameter									Random effect						
		*α*_1_									Standard deviation						
		Estimate	SE	*p* value							*Site*_1*k*_	*Culm*_1*jk*_	*e*_*ijk*_				
TM	I	23.075	0.546	< 0.001							–	2.287	5.167				

Property	Eq	*γ*_0_			*γ*_1_						Standard deviation						
		Estimate	SE	*p *value	Estimate	SE	*p* value				*Site*_0*k*_	*Culm*_0*jk*_	*Site*_1*k*_	*Culm*_1*jk*_	*e*_*ijk*_		
FL	III	− 0.577	0.064	< 0.001	3.931	0.052	< 0.001				0.105	0.085	–	–	0.217		
CS	III	4.966	1.163	< 0.001	60.733	2.69	< 0.001				–	4.151	–	14.065	6.324		
TS	III	− 10.678	6.859	0.122	242.529	11.331	< 0.001				–	–	–	21.469	45.168		

Property	Eq	*ζ*_0_			*ζ*_1_			*ζ*_2_			Standard deviation						
		Estimate	SE	*p *value	Estimate	SE	*p* value	Estimate	SE	*p *value	*Site*_0*k*_	*Culm*_0*jk*_	*Site*_1*k*_	*Culm*_1*jk*_	*Site*_2*k*_	*Culm*_2*jk*_	*e*_*ijk*_
FA	IV	− 0.376	0.103	< 0.001	3.501	1.091	0.002	40.52	2.852	< 0.001	–	0.312	–	3.703	–	12.745	5.73
MOE	IV	− 0.249	0.041	< 0.001	2.649	0.414	< 0.001	8.789	1.074	< 0.001	–	–	–	–	1.043	1.508	2.062
MOR	IV	− 1.342	0.326	< 0.001	14.988	3.326	< 0.001	141.839	6.245	< 0.001	–	1.056	–	10.858	–	–	14.188

*FL* fiber length; FA, fiber area; MOE, modulus of elasticity; MOR, modulus of rupture; CS, compressive strength parallel to grain; TM, tensile Young’s modulus; TS, tensile strength; Eq., fitted model; SE, standard error; *α*_1_, *γ*_0_, *γ*_1_, *ζ*_0_, *ζ*_1_, and *ζ*_2_, the fixed effects; *Site*_0*k*_, *Site*_1*k*_, and *Site*_2*k*_*,* the random effect at the site level; *Culm*_0*jk*_, *Culm*_1*jk*_, and *Culm*_2*jk*_ the random effect at the individual culm level; *e*_*ijk*_, residual.

**Figure 5:**
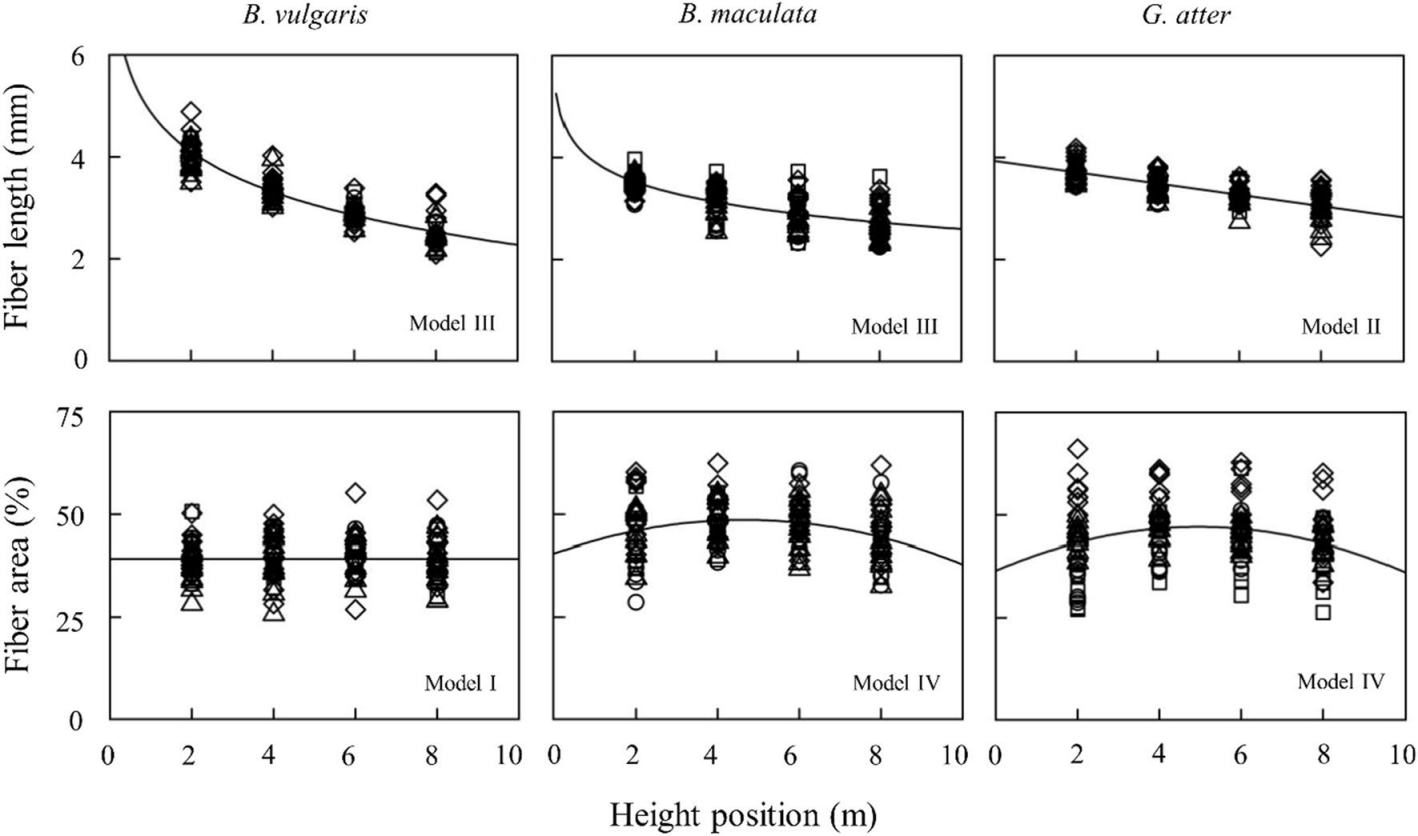
Longitudinal variations of anatomical characteristics in three bamboo species. Note: Number of samples = 40 individual culms. The regression lines or curves are based on the fixed-effect parameters of the most parsimonious models with minimum AIC among the developed models listed in Tables S2, S3, S4. The symbols of circles, triangles, squares, and diamonds in each figure indicate Site I, II, III, and IV, respectively. The graph was originally created by R^27^ (version 4.0.3, https://www.R-project.org/).

**Figure 6:**
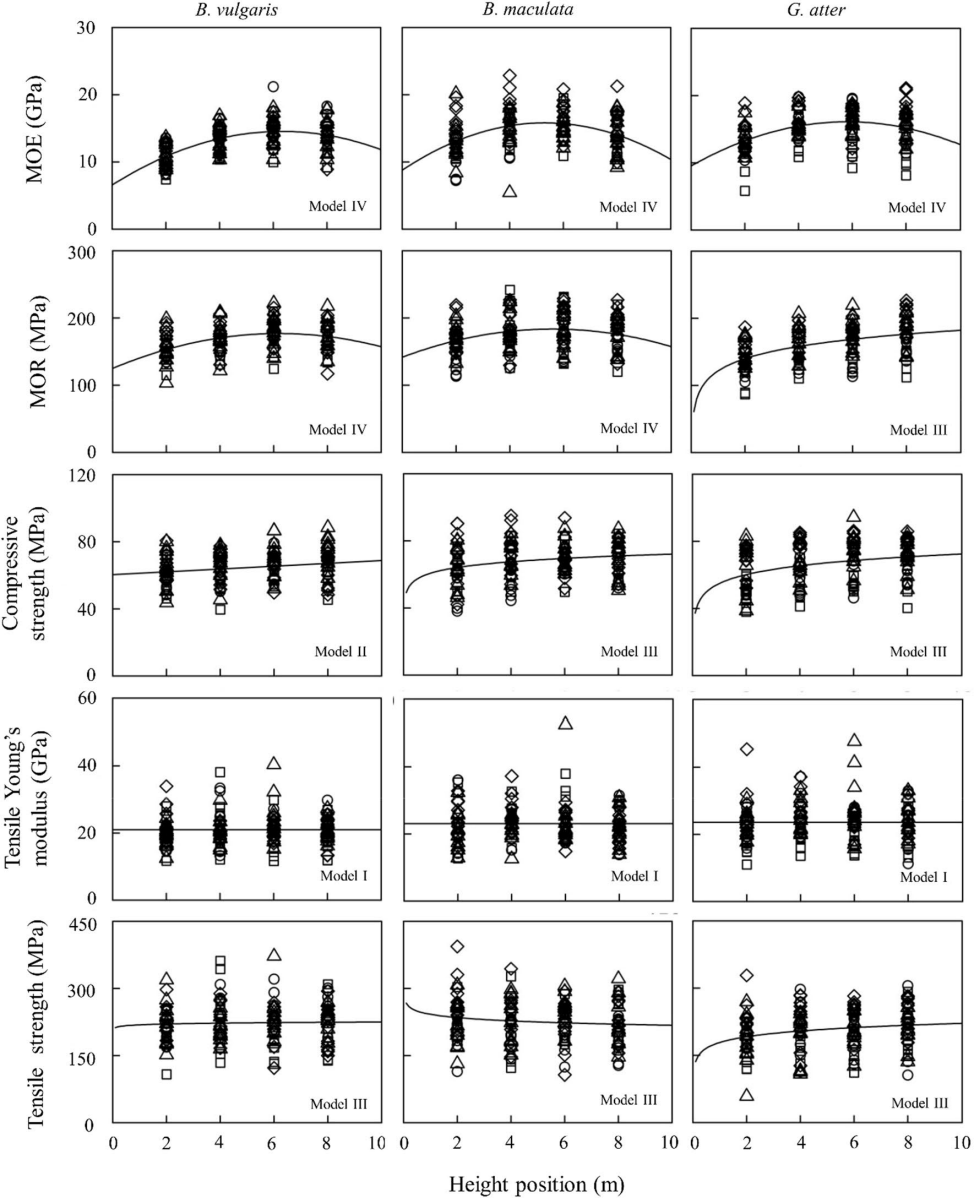
Longitudinal variations of mechanical properties in three bamboo species. Note: Number of samples = 40 individual culms. The regression lines or curves are based on the fixed-effect parameters of the most parsimonious models with minimum AIC among the developed models listed in Tables S2, S3, S4. The symbols of circles, triangles, squares, and diamonds in each figure indicate Site I, II, III, and IV, respectively. The graph was originally created by R^27^ (version 4.0.3, https://www.R-project.org/).

The AIC values of developed models for longitudinal variation of anatomical characteristics and mechanical properties in three bamboo species are listed in Tables S2 to S4. Although 36 models were developed in the present study, only the converge models were included in Tables S2, S3, S4. Based on the results of AIC value, TM of all species and fiber area of *B. vulgaris* were fitted to the *y*-intercept model (Model I), CS of *B. vulgaris* and fiber length of *G. atter* to a linear mixed-effects model (Model II), whereas nonlinear mixed-effects models (logarithmic and quadratic equation, Models III and IV) were adapted to the other properties in three bamboo species. Figures 5 and 6 show the longitudinal variation of culm properties. The lines or curves in the figures were fixed-effect parameters in selected models (Tables 3 , 4, 5). Table 6 shows the classification of longitudinal variations of culm properties according to Fig. 4. Fiber area and TM were stable from base to top in all species. In addition, the CS and TS of *Bambusa* species also showed a stable tendency. Fiber length decreased from base to top in all species, whereas MOE and MOR increased from base to top in all species except for MOR in *B. maculata*. In addition, a similar tendency (increasing values from base to top) was found in CS and TS of *G. atter*.

**Table 5:** Parameter estimates, standard errors, and *p* values of fixed-effect parameters, and standard deviations of the random effect estimates for the most parsimonious models of culm properties in *G.atter.*

Property	Eq	Fixed-effect parameter									Random effect						
		*α*_1_									Standard deviation						
		Estimate	SE	*p* value							*Site*_1*k*_	*Culm*_1*jk*_	*e*_*ijk*_				
TM	I	23.620	1.523	< 0.001							2.091	2.866	5.03				

Property	Eq	*β*_0_			*β*_1_						Standard deviation						
		Estimate	SE	*p* value	Estimate	SE	*p* value				*Site*_0*k*_	*Culm*_0*j*_	*Site*_1*k*_	*Culm*_1*jk*_	*e*_i*jk*_		
FL	II	− 0.111	0.007	< 0.001	3.939	0.035	< 0.001				–	0.023	–	–	0.182		

Property	Eq	*γ*_0_			*γ*_1_						Standard deviation						
		Estimate	SE	*p* value	Estimate	SE	*p* value				*Site*_0*k*_	*Culm*_0*jk*_	*Site*_1*k*_	*Culm*_1*jk*_	*e*_*ijk*_		
MOR	III	26.314	2.001	< 0.001	121.799	8.219	< 0.001				–	–	14.327	15.887	13.176		
CS	III	7.643	0.823	< 0.001	54.925	3.811	< 0.001				–	–	6.694	8.108	5.421		
TS	III	18.494	6.477	0.005	178.985	13.436	< 0.001				–	–	16.951	13.345	42.656		

Property	Eq	*ζ*_0_			*ζ*_1_			*ζ*_2_			Standard deviation						
		Estimate	SE	*p* value	Estimate	SE	*p* value	Estimate	SE	*p* value	*Site*_0*k*_	*Culm*_0*jk*_	*Site*_1*k*_	*Culm*_1*jk*_	*Site*_2*k*_	*Culm*_2*jk*_	*e*_*ijk*_
FA	IV	− 0.432	0.095	< 0.001	4.278	0.965	< 0.001	36.518	3.089	11.821	–	–	–	–	4.357	3.606	4.805
MOE	IV	− 0.194	0.033	< 0.001	2.259	0.333	< 0.001	9.552	1.012	< 0.001	–	–	–	–	1.317	1.512	1.659

*FL* fiber length; *FA* fiber area; *MOE* modulus of elasticity; *MOR* modulus of rupture; *CS* compressive strength parallel to grain; *TM* tensile Young’s modulus; TS, tensile strength; Eq., fitted model; SE, standard error; *α*_1_, *β*_0_, *β*_1_, *γ*_0_, *γ*_1_, *ζ*_0_, *ζ*_1_, and *ζ*_2_, the fixed effects; *Site*_0*k*_, *Site*_1*k*_, and *Site*_2*k*_*,* the random effect at the site level; *Culm*_0*jk*_, *Culm*_1*jk*_, and *Culm*_2*jk*_ the random effect at the individual culm level; *e*_*ijk*_, residual.

**Figure 7:**
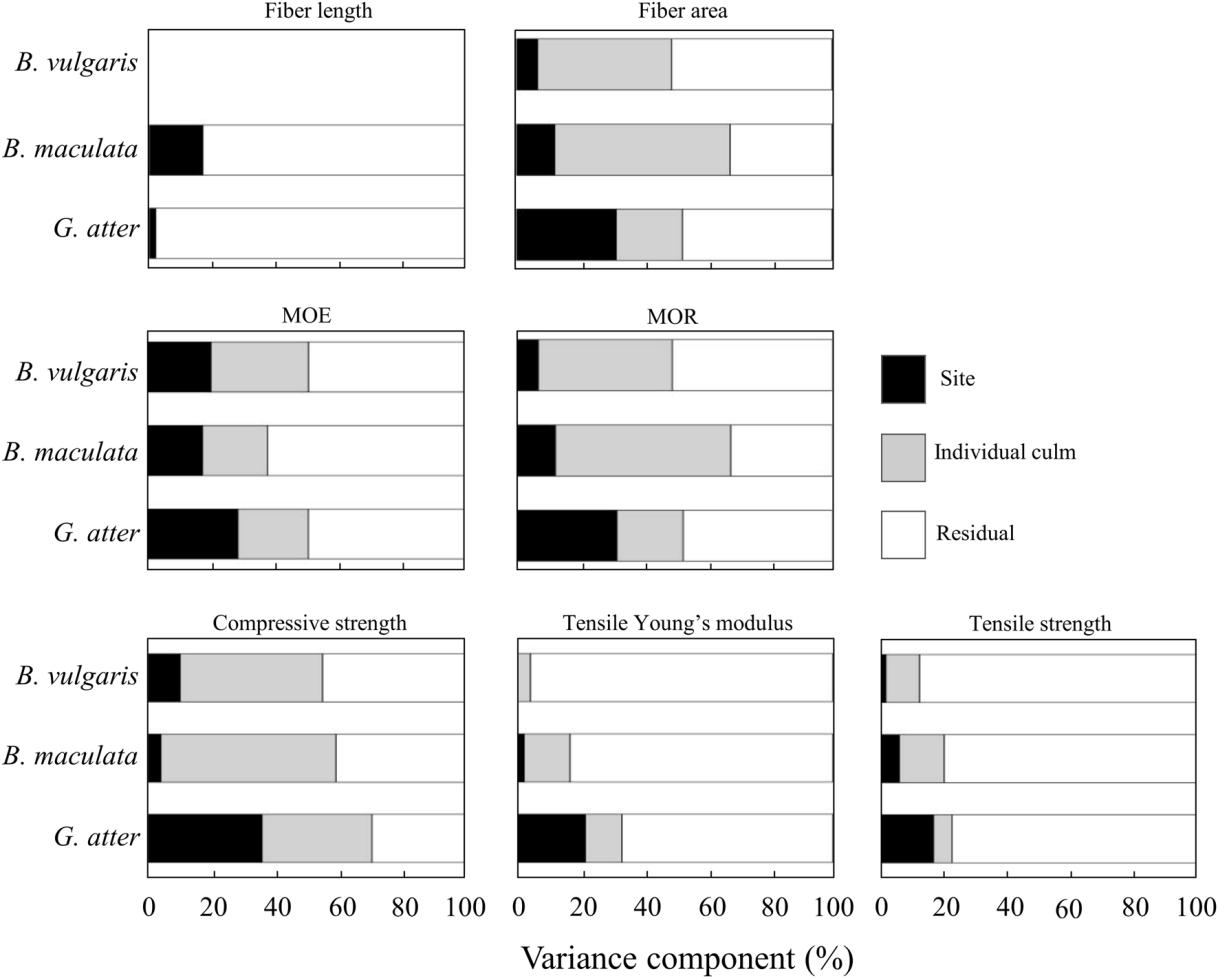
Variance components of site and individual culm as random effects in the intercept only linear mixed-effect model of anatomical characteristics and mechanical properties in three bamboo species. Note: The model used to determine the variance components for fiber length of *B. vulgaris* was failed to converge. The graph was originally created by R^27^ (version 4.0.3, https://www.R-project.org/).

Figure 7 shows the variance components of the site and individual culm as random effects in the intercept-only liner-mixed effects models for anatomical characteristics and mechanical properties. The site variance was found in all properties in all species, except for fiber length and TM of *B. vulgaris*, but their values were less than 40%. The larger values of site variance components were found in *G. atter* compared to those in the other two *Bambusa* species. Table 7 shows random-effect parameter estimates of the site in each property. In *G. atter*, random-effect parameter estimates were larger values in site IV and lower values in site III. In *B. maculata*, the higher values were also found in site IV, but the lower values were recognized in site I. On the other hand, a distinct tendency was not observed in *B. vulgaris*.

**Table 6:** Classification of longitudinal variation patterns in bamboo culm properties based on the results of mixed-effect modelling.

Property	Species	Model	Minimum	Maximum	CV (%)	Type
FL (mm)	BV	III	2.53 (8.0 m)	4.11 (2.0 m)	14.3	C-i
	BM	III	2.73 (8.0 m)	3.53 (2.0 m)	7.6	C-i
	GA	II	3.05 (8.0 m)	3.72 (2.0 m)	5.9	B-i
FA (%)	BV	I	–	–	0.2	A
	BM	IV	44.5 (8.0 m)	48.7 (4.9 m)	2.4	A
	GA	IV	43.1 (2.0 m)	47.1 (8.0 m)	2.6	A
MOE (GPa)	BV	IV	10.84 (2.0 m)	14.56 (5.8 m)	8.0	D-ii
	BM	IV	13.09 (2.0 m)	15.83 (5.3 m)	5.0	D-ii
	GA	IV	13.29 (2.0 m)	16.13 (6.3 m)	5.1	D-ii
MOR (MPa)	BV	IV	153.2 (2.0 m)	177.0 (6.2 m)	4.0	D-ii
	BM	IV	166.5 (2.0 m)	183.7 (5.6 m)	2.6	A
	GA	III	140.0 (2.0 m)	176.5 (8.0 m)	6.4	C-ii
CS (MPa)	BV	II	62.2 (2.0 m)	67.2 (8.0 m)	2.3	A
	BM	III	64.2 (2.0 m)	71.1 (8.0 m)	2.9	A
	GA	III	60.2 (2.0 m)	70.8 (8.0 m)	4.5	C-ii
TM (GPa)	BV	I	–	–	0.8	A
	BM	I	–	–	0.2	A
	GA	I	–	–	0.9	A
TS (MPa)	BV	III	220.89 (8.0 m)	224.7 (2.0 m)	0.5	A
	BM	III	220.3 (2.0 m)	235.1 (8.0 m)	1.9	A
	GA	III	191.8 (2.0 m)	217.4 (8.0 m)	3.5	C-ii

*FL* fiber length; *FA* fiber area; *MOE* modulus of elasticity; *MOR* modulus of rupture; *CS* compressive strength parallel to the grain; *TM* tensile Young’s modulus; *TS* tensile strength; *BV B. vulgaris*; *BM B. maculata*; *GA G. atter*; *CV* coefficient of variation, Model I, II, III, and IV are based on intercept only, linear, logarithmic, and quadratic formulae, respectively. Minimum and maximum values are estimated from 2.0 to 8.0 m above the ground using fixed-effect parameters of the selected mixed-effect models (Tables 3 , 4 , 5). Bars in minimum and maximum values indicate no available data because intercept only model was selected. Values in parentheses after minimum and maximum values indicate height positions showing minimum and maximum values. Type refers to Fig. 4.

**Table 7:** Parameter estimates, standard errors, and *p* values of the selected models for geographic variations of bamboo culm properties.

Species	Property	Fixed-effect parameter			Random-effect parameter estimates			
		Estimates	SE	*p* value	Site I	Site II	Site III	Site IV
BV	FL	–	–	–	–	–	–	–
	FA	39.324	0.622	< 0.001	0.228	− 0.365	− 0.225	0.362
	MOE	13.219	0.515	< 0.001	0.826	0.245	− 1.082	0.010
	MOR	168.338	4.007	< 0.001	0.545	1.652	− 6.173	3.976
	CS	64.667	1.868	< 0.001	− 0.438	3.227	− 2.542	− 0.246
	TM	21.016	0.398	< 0.001	–	–	–	–
	TS	223.038	4.804	< 0.001	2.577	0.997	− 2.912	− 0.661
BM	FL	3.073	0.093	< 0.001	− 0.213	− 0.045	0.111	0.148
	FA	46.730	1.335	< 0.001	− 2.035	− 0.076	− 0.879	2.990
	MOE	14.564	0.682	< 0.001	− 0.677	− 0.526	− 0.478	1.681
	MOR	176.527	6.177	< 0.001	− 10.617	1.781	− 0.083	8.919
	CS	68.121	1.869	< 0.001	− 1.789	0.367	− 0.129	1.552
	TM	23.075	0.684	< 0.001	− 0.465	− 0.456	0.346	0.575
	TS	226.644	7.497	< 0.001	− 13.439	1.927	5.890	5.622
GA	FL	3.383	0.035	< 0.001	− 0.013	− 0.039	0.032	0.198
	FA	44.948	2.612	< 0.001	− 2.171	− 0.247	− 4.425	6.844
	MOE	15.022	0.815	< 0.001	0.173	0.236	− 1.959	1.549
	MOR	160.946	8.791	< 0.001	− 11.985	12.063	− 15.392	15.314
	CS	66.296	4.142	0.001	− 3.934	3.222	− 7.970	8.684
	TM	23.620	1.523	0.001	0.111	1.691	− 3.843	2.041
	TS	206.500	10.740	< 0.001	− 1.080	− 11.369	− 14.114	26.564

*SE* standard error; *BV B. vulgaris*; *BM B. maculata*; *GA G. atter*; *FL* fiber length; *FA* fiber area; *MOE* modulus of elasticity; *MOR* modulus of rupture; *CS* compressive strength parallel to the grain; *TM* tensile Young’s modulus; *TS* tensile strength; –, model was not converged.

## Discussion

### Anatomical characteristics and mechanical properties of bamboo culm

The average fiber lengths of *B. vulgaris* and *B. maculata* in the present study were within the range of the values reported by previous researchers for some *Bambusa* species^6,9,34^, whereas fiber lengths in *G. atter* used this study were higher than those obtained in previous research on the same species^13^ and *G. scortechinii* grown in Malaysia^9^. The MOE, MOR, and CS values of *Bambusa* species obtained in the present study were lower than *B.rigida*^11^, but the MOE and MOR were higher than *B. blumeana* and *B. heterostachya*^9^, and the TM of *B. maculata* was higher than *B. balcoa*^8^. The average values of MOE and TM in *G. atter* obtained in the present study were higher than those previously reported in some *Gigantochloa* species^9,12,15^, while the CS and TS value were lower than *G. scortechinii* and *G. levis*^12,15^.

### Longitudinal variations

Based on the results in Tables 3, 4, 5, the best model for explaining longitudinal variation of some anatomical and mechanical properties in *B. vulgaris* (TS), *B. maculata* (fiber length, fiber area, MOR, and CS), and *G. atter* (fiber length) included the random effects of individual culms in slope, suggesting that the slope of the line or curve on these properties varies between individual culms within the species. However, because the coefficient of variation of TS in *B. vulgaris*, fiber area and CS in *B. maculata* were less than 3.0% (Type A in Table 6), the differences of slope among individual culms in these properties could be ignored. The longitudinal variations of other bamboo properties except for fiber length in *B. vulgaris* fitted the model with random effects of individual culm in the intercept. These results suggested that the mean values of some bamboo properties in a longitudinal direction vary between individual culms within the species, and the longitudinal patterns may be similar between individual culms. In addition, the best longitudinal model of fiber length in *B. vulgaris*, MOE in *B. maculata*, and all properties (except for fiber length) in *G. atter* included random effects of sites in the intercept. These results indicated that these properties also vary between sites. The influence of sites on bamboo properties such as green moisture content and basic density was also found in our previous studies^23^.

Longitudinal variations of culm properties in *B. vulgaris* were similar to those of *B. maculata* (Figs. 5 and 6, Table 6), while in *G.atter* were not similar to those of the other two *Bambusa* species. Thus, it is considered that longitudinal variations of culm properties may depend on genus level. In *Bambusa* species, almost all culm properties were stable from base to top, but some properties, such as fiber length, MOE, and MOR (except for *B. maculata*) varied from base to top. Compared to *Bambusa* species, *G. atter* had longitudinal variations in many culm properties: many mechanical properties showed lower values on the base side and higher values on the top side.

### Geographic variations

Previously, we evaluated the geographic variations of green moisture content and basic density in the same species by mixed-effects modeling^23^. We found that *G. atter* had the larger variance components of the sites in both properties than those in the *Bambusa* species, suggesting that green moisture content and basic density of *G. atter* have the larger geographic variations^23^. In the present study, similar results were obtained in other properties, i.e. fiber area, MOE, MOR, CS, TM, and TS (Fig. 7). Thus, it is considered that the site variations of anatomical characteristics and mechanical properties may differ among species: *G. atter* has larger geographic variations, but not so much in two *Bambusa* species. On the other hand, the variance component of individual culm was larger than sites in *B. vulgaris* and *B. maculata* (Fig. 7), indicating that each property differs between individual culms within a site for these two species. The bamboo used in the present study was collected from the natural population in each site, assuming that genetic backgrounds may differ among the sites in each species. Because the genus of *Bambusa* is polyphyletic, it typically displays a high level of genetic diversity^35,36^. Thus, the differences may occur in individual properties within *Bambusa* species in a population at a site. The high variation of individual culm in two *Bambusa* species in the present study may be due to their evolutionary history and geological development^37^. High genetic diversity was also detected in* D. membranaceus* from 12 natural populations in Yunnan, China^37^. It was reported that most of the genetic variation (78.95%) was among individuals in the population, whereas only 21.05% existed among the population^37^. Thus, anatomical and mechanical properties of culm in some bamboo species may have a larger variation of individuals compared to that within a site. On the other hand, *G. atter* had a relatively larger site variation than that of the other two *Bambusa* species. Generally, *G. atter* can propagate vegetatively through rhizome or stem cuttings^22^, leading to the smaller variation of culm properties within a population. In addition, the natural population of each species at each location is related to its environmental conditions^38^. Environmental factors, particularly precipitation, influence bamboo growth significantly^3,39^. *G. atter* can grow well in the habitats with more than 2,500 mm year^−1^ of precipitation^22^. In the present study, annual precipitation at the four sites ranged from 705 to 2,464 mm year^−1^ (Fig. 1), suggesting that growth of this species may be regulated in lower precipitation site such as site III (705 mm year^−1^). In fact, culm diameter, height, and thickness of *G. atter* showed the lowest values in site III (Table 1)^23^. Therefore, we concluded that 1) large among-individual variations within a natural population were found in *Bambusa* species, 2) among-individual variations within a population in *G. atter* is small, and 3) difference in the amount of precipitation might cause larger site variation in *G. atter* compare to two *Bambusa* species.

## Conclusions

In this study, the *y*-intercept, the linear mixed-effects, and the nonlinear mixed-effects models (logarithmic and quadratic equations) were used to evaluate the longitudinal variation of the anatomical characteristics and mechanical properties of three bamboo species naturally growing in four different sites of Lombok Island, Indonesia. The longitudinal variation in the two *Bambusa* species showed the similar patterns in many examined properties: fiber length and MOE varied from the base to the top, while other properties were stable from the bottom to the top of the culm. In *G. atter*, almost all longitudinal variations in mechanical properties showed lower values on the bottom side and higher values on the top side. Geographic variations of bamboo culm properties were also evaluated by the mixed-effects model. As the results, the variance component of individual culms was higher than that of sites in *B. vulgaris* and *B. maculata*, indicating that each property differs between individual culms within the site for these two species. On the other hand, *G. atter* had a higher variance component of the site than the other two *Bambusa* species. In *G. atter*, the estimated random-effect parameter was higher at site IV and lower at site III. These differences might be related to the differences of precipitation in these sites. Based on the results, we concluded that effective utilization of bamboo culm for modern construction materials is possible for two *Bambusa* species because almost all properties showed stable from base to top, but variation of individual culm rather than site should be considered. On the other hand, when bamboo culm of *G. atter* was used for modern construction materials, longitudinal variation of mechanical properties and site variation should be considered.

## Supplementary Information

Supplementary Information.

## Data Availability

The dataset generated during and/or analysed during the current study are available in the fig share repository, https://doi.org/10.6084/m9.figshare.21521214.
