# Supplementary material for: Geographic and longitudinal variations of anatomical characteristics and mechanical properties in three bamboo species naturally grown in Lombok Island, Indonesia
Source: Sci Rep. 2023 Feb 8;13:2265. doi: 10.1038/s41598-023-29288-3 (PMC9908941; doi:10.1038/s41598-023-29288-3)
Supplement: Supplementary file 1 — Supplementary Information. [file 41598_2023_29288_MOESM1_ESM.docx]

**Table S1**. The air-dry density and moisture content of specimen at mechanical testing.

| **Species** | **Bending test specimen** | | **Compressive test specimen** | | **Tensile test specimen** | |
| --- | --- | --- | --- | --- | --- | --- |
|  | **AD (g cm^−3^)** | **MC (%)** | **AD (g cm^−3^)** | **MC (%)** | **AD (g cm^−3^)** | **MC (%)** |
| *B. vulgaris* | 0.72 | 10.2 | 0.73 | 11.3 | 0.73 | 6.8 |
| *B. maculata* | 0.73 | 10.1 | 0.73 | 10.9 | 0.73 | 6.5 |
| *G. atter* | 0.71 | 10.1 | 0.70 | 11.3 | 0.71 | 6.5 |

Note: AD, air-dry density; MC, moisture content.

**Table S2.** Comparison of AIC values in developed linear and nonlinear mixed-effects models for longitudinal variation of anatomical characteristics and mechanical properties in *B. vulgaris*

| **Model rank** | **Model Eq.** | **Random effect** | | | | **AIC** | **ΔAIC** | **Model Eq.** | **Random effect** | | | | **AIC** | **ΔAIC** |
| --- | --- | --- | --- | --- | --- | --- | --- | --- | --- | --- | --- | --- | --- | --- |
|  |  | ***Site*_0_** | ***Culm*_0_** | ***Site*_1_** | ***Culm*_1_** |  |  |  | ***Site*_0_** | ***Culm*_0_** | ***Site*_1_** | ***Culm*_1_** |  |  |
|  | FL | | | | | | | FA | | | | | | |
| 1^st^ | **III** |  |  | **+** |  | **10.112** | **0** | **I** |  |  |  | **+** | **972.452** | **0** |
| 2^nd^ | III |  |  |  | + | 10.843 | 0.731 | III |  | + |  |  | 973.134 | 0.682 |
| 3^rd^ | III |  | + |  |  | 11.109 | 0.997 | III |  |  |  | + | 973.294 | 0.842 |
| 4^th^ | III | + |  |  |  | 11.222 | 1.110 | I |  |  | + | + | 974.287 | 1.835 |
| 5^th^ | III |  | + |  | + | 11.391 | 1.279 | III | + | + |  |  | 975.134 | 2.682 |
| 6^th^ | III |  |  | + | + | 11.828 | 1.716 | III |  |  | + | + | 975.248 | 2.796 |
| 7^th^ | III | + | + |  |  | 12.906 | 2.794 | II |  |  |  | + | 976.286 | 3.834 |
| 8^th^ | IV |  |  | + |  | 18.621 | 8.509 | II |  | + |  |  | 977.402 | 4.950 |
| 9^th^ | IV |  |  |  | + | 19.178 | 9.066 | II |  | + |  | + | 977.563 | 5.111 |
| 10^th^ | IV |  |  | + | + | 20.164 | 10.052 | II |  |  | + | + | 978.117 | 5.665 |
| 11^th^ | II |  |  | + |  | 20.613 | 10.501 | II | + |  |  | + | 978.261 | 5.809 |
| 12^th^ | IV |  | + |  |  | 21.513 | 11.401 | II |  | + | + |  | 978.846 | 6.394 |
| 13^th^ | II | + |  |  |  | 21.526 | 11.414 | II | + | + |  |  | 979.348 | 6.896 |
| 14^th^ | IV |  | + |  | + | 21.666 | 11.554 | II |  | + | + | + | 979.360 | 6.908 |
| 15^th^ | II |  |  |  | + | 21.673 | 11.561 | II | + | + |  | + | 979.559 | 7.107 |
| 16^th^ | II |  | + |  |  | 21.794 | 11.682 | IV |  |  |  | + | 979.612 | 7.160 |
| 17^th^ | II |  | + | + |  | 22.501 | 12.389 | IV |  |  | + | + | 981.567 | 9.115 |
| 18^th^ | II |  |  | + | + | 22.534 | 12.422 | I |  |  | + |  | 982.032 | 9.580 |
| 19^th^ | II | + |  |  | + | 23.361 | 13.249 | IV |  | + |  |  | 982.585 | 10.133 |
| 20^th^ | II | + | + |  |  | 23.405 | 13.293 | III |  |  | + |  | 982.771 | 10.319 |
| 21^st^ | II |  | + |  | + | 23.662 | 13.550 | III | + |  |  |  | 983.310 | 10.858 |
| 22^nd^ | IV | + |  |  |  | 23.677 | 13.565 | II |  |  | + |  | 985.622 | 13.170 |
| 23^rd^ | II |  | + | + | + | 24.498 | 14.386 | II | + |  |  |  | 986.159 | 13.707 |
| 24^th^ | II | + | + |  | + | 25.354 | 15.242 | IV |  |  | + |  | 989.187 | 16.735 |
| 25^th^ | IV | + | + |  |  | 27.249 | 17.137 |  |  |  |  |  |  |  |
|  | MOE | | | | | | | MOR | | | | | | |
| 1^st^ | **IV** |  |  | **+** | **+** | **627.668** | **0** | **IV** |  |  |  | **+** | **1356.296** | **0** |
| 2^nd^ | IV |  | + |  |  | 630.216 | 2.548 | IV |  |  | + | + | 1357.355 | 1.059 |
| 3^rd^ | III |  | + |  | + | 644.616 | 16.948 | IV |  | + |  |  | 1359.008 | 2.712 |
| 4^th^ | III |  |  | + | + | 644.700 | 17.032 | IV | + | + |  |  | 1362.850 | 6.554 |
| 5^th^ | III | + | + | + | + | 647.949 | 20.281 | III |  | + |  | + | 1365.018 | 8.722 |
| 6^th^ | III | + | + |  |  | 648.129 | 20.461 | III |  |  |  | + | 1365.733 | 9.437 |
| 7^th^ | II |  | + | + | + | 670.275 | 42.607 | III |  |  | + | + | 1366.791 | 10.495 |
| 8^th^ | II |  |  | + | + | 670.508 | 42.840 | III | + | + | + | + | 1368.607 | 12.311 |
| 9^th^ | II | + |  |  | + | 671.899 | 44.231 | II | + |  |  | + | 1378.813 | 22.517 |
| 10^th^ | II | + |  | + | + | 672.108 | 44.440 | III |  | + |  |  | 1379.151 | 22.855 |
| 11^th^ | II | + | + | + | + | 672.136 | 44.468 | II |  |  |  | + | 1379.199 | 22.903 |
| 12^th^ | II | + | + |  | + | 672.633 | 44.965 | III | + | + |  |  | 1380.130 | 23.834 |
| 13^th^ | II |  | + |  | + | 673.003 | 45.335 | II |  |  | + | + | 1380.133 | 23.837 |
| 14^th^ | II |  | + | + |  | 673.164 | 45.496 | II | + |  | + | + | 1380.137 | 23.841 |
| 15^th^ | II |  |  |  | + | 673.234 | 45.566 | II | + | + |  | + | 1380.156 | 23.860 |
| 16^th^ | II | + | + | + |  | 675.163 | 47.495 | II |  | + | + | + | 1380.574 | 24.278 |
| 17^th^ | II | + | + |  |  | 677.783 | 50.115 | II | + | + | + | + | 1381.245 | 24.949 |
| 18^th^ | IV |  |  | + |  | 678.188 | 50.52 | II |  | + | + |  | 1396.026 | 39.730 |
| 19^th^ | II |  | + |  |  | 680.013 | 52.345 | II | + | + | + |  | 1397.974 | 41.678 |
| 20^th^ | III | + |  |  |  | 683.768 | 56.100 | II |  | + |  |  | 1398.955 | 42.659 |
| 21^st^ | II |  |  | + |  | 698.382 | 70.714 | II | + | + |  |  | 1399.823 | 43.527 |
| 22^nd^ | II | + |  | + |  | 700.352 | 72.684 | I |  |  |  | + | 1413.247 | 56.951 |
| 23^rd^ | II | + |  |  |  | 700.966 | 73.298 | I |  |  | + | + | 1414.181 | 57.885 |
| 24^th^ | I |  |  | + | + | 732.015 | 104.350 | III |  |  | + |  | 1423.170 | 66.874 |
| 25^th^ | I |  |  |  | + | 734.741 | 107.070 | III | + |  |  |  | 1423.836 | 67.540 |
| 26^th^ | I |  |  | + |  | 739.736 | 112.070 | III | + |  | + |  | 1427.006 | 70.710 |
| 27^th^ |  |  |  |  |  |  |  | II |  |  | + |  | 1430.815 | 74.519 |
| 28^th^ |  |  |  |  |  |  |  | II | + |  |  |  | 1432.186 | 75.890 |
| 29^th^ |  |  |  |  |  |  |  | II | + |  | + |  | 1432.646 | 76.350 |
| 30^th^ |  |  |  |  |  |  |  | I |  |  | + |  | 1448.466 | 92.170 |

Table S2. Continued

| **Model rank** | **Model Eq.** | **Random effect** | | | | **AIC** | **ΔAIC** | **Model Eq.** | **Random effect** | | | | **AIC** | **ΔAIC** |
| --- | --- | --- | --- | --- | --- | --- | --- | --- | --- | --- | --- | --- | --- | --- |
|  |  | ***Site*_0_** | ***Culm*_0_** | ***Site*_1_** | ***Culm*_1_** |  |  |  | ***Site*_0_** | ***Culm*_0_** | ***Site*_1_** | ***Culm*_1_** |  |  |
|  | CS | | | | | | | TM | | | | | | |
| 1^st^ | **II** | **+** |  |  | **+** | **1094.029** | **0** | III |  | + |  |  | 956.542 | 0 |
| 2^nd^ | II | + |  | + | + | 1095.224 | 1.195 | **I** |  |  |  | **+** | **956.547** | **0.005** |
| 3^rd^ | III |  |  |  | + | 1102.194 | 8.165 | III | + |  |  |  | 956.731 | 0.189 |
| 4^th^ | III |  |  | + | + | 1102.391 | 8.362 | III |  |  |  | + | 957.058 | 0.516 |
| 5^th^ | III |  | + |  | + | 1102.528 | 8.499 | III |  |  | + |  | 957.437 | 0.895 |
| 6^th^ | II |  |  |  | + | 1106.054 | 12.025 | III | + | + |  |  | 958.215 | 1.673 |
| 7^th^ | II |  |  | + | + | 1106.127 | 12.098 | III |  |  | + | + | 959.058 | 2.516 |
| 8^th^ | II |  | + |  | + | 1106.238 | 12.209 | II | + |  |  |  | 959.421 | 2.879 |
| 9^th^ | II |  | + | + | + | 1107.298 | 13.269 | II |  | + |  |  | 959.991 | 3.449 |
| 10^th^ | IV |  |  |  | + | 1108.906 | 14.877 | II |  |  |  | + | 960.254 | 3.712 |
| 11^th^ | IV |  |  | + | + | 1109.103 | 15.074 | II | + |  |  | + | 961.068 | 4.526 |
| 12^th^ | III | + | + |  |  | 1111.732 | 17.703 | II | + | + |  |  | 961.181 | 4.639 |
| 13^th^ | III |  | + |  |  | 1112.885 | 18.856 | II |  | + |  | + | 961.991 | 5.449 |
| 14^th^ | IV |  | + |  |  | 1114.786 | 20.757 | IV |  |  |  | + | 962.128 | 5.586 |
| 15^th^ | IV |  | + |  | + | 1114.839 | 20.810 | IV |  |  | + |  | 962.586 | 6.044 |
| 16^th^ | I |  |  |  | + | 1117.470 | 23.441 | IV |  |  | + | + | 964.128 | 7.586 |
| 17^th^ | I |  |  | + | + | 1117.489 | 23.460 |  |  |  |  |  |  |  |
| 18^th^ | II | + | + | + |  | 1122.212 | 28.183 |  |  |  |  |  |  |  |
| 19^th^ | II | + | + |  |  | 1122.496 | 28.467 |  |  |  |  |  |  |  |
| 20^th^ | II |  | + | + |  | 1123.343 | 29.314 |  |  |  |  |  |  |  |
| 21^st^ | II |  | + |  |  | 1124.314 | 30.285 |  |  |  |  |  |  |  |
| 22^nd^ | III | + |  |  |  | 1148.356 | 54.327 |  |  |  |  |  |  |  |
| 23^rd^ | III | + |  | + |  | 1150.844 | 56.815 |  |  |  |  |  |  |  |
| 24^th^ | II | + |  |  |  | 1151.197 | 57.168 |  |  |  |  |  |  |  |
| 25^th^ | II | + |  | + |  | 1152.844 | 58.815 |  |  |  |  |  |  |  |
| 26^th^ | II |  |  | + |  | 1154.897 | 60.868 |  |  |  |  |  |  |  |
| 27^th^ | IV |  |  | + |  | 1157.959 | 63.930 |  |  |  |  |  |  |  |
| 28^th^ | I |  |  | + |  | 1159.680 | 65.651 |  |  |  |  |  |  |  |
|  | TS | | | | | | |  |  |  |  |  |  |  |
| 1^st^ | **III** |  | **+** |  |  | **1673.135** | **0** |  |  |  |  |  |  |  |
| 2^nd^ | III |  |  |  | + | 1673.601 | 0.466 |  |  |  |  |  |  |  |
| 3^rd^ | III | + | + |  |  | 1674.731 | 1.596 |  |  |  |  |  |  |  |
| 4^th^ | III | + |  |  |  | 1674.958 | 1.823 |  |  |  |  |  |  |  |
| 5^th^ | III |  |  | + |  | 1675.825 | 2.690 |  |  |  |  |  |  |  |
| 6^th^ | II |  | + |  |  | 1676.439 | 3.304 |  |  |  |  |  |  |  |
| 7^th^ | II |  |  |  | + | 1676.618 | 3.483 |  |  |  |  |  |  |  |
| 8^th^ | IV |  |  |  | + | 1676.632 | 3.497 |  |  |  |  |  |  |  |
| 9^th^ | III |  | + |  | + | 1676.823 | 3.688 |  |  |  |  |  |  |  |
| 10^th^ | I |  |  |  | + | 1677.379 | 4.244 |  |  |  |  |  |  |  |
| 11^th^ | II | + |  |  | + | 1677.613 | 4.478 |  |  |  |  |  |  |  |
| 12^th^ | II | + |  |  |  | 1677.732 | 4.597 |  |  |  |  |  |  |  |
| 13^th^ | II | + | + |  |  | 1677.814 | 4.679 |  |  |  |  |  |  |  |
| 14^th^ | II |  | + |  | + | 1678.147 | 5.012 |  |  |  |  |  |  |  |
| 15^th^ | II |  | + | + |  | 1678.411 | 5.276 |  |  |  |  |  |  |  |
| 16^th^ | II |  |  | + | + | 1678.500 | 5.365 |  |  |  |  |  |  |  |
| 17^th^ | IV |  |  | + | + | 1678.616 | 5.481 |  |  |  |  |  |  |  |
| 18^th^ | II |  |  | + |  | 1678.708 | 5.573 |  |  |  |  |  |  |  |
| 19^th^ | IV |  |  | + |  | 1678.783 | 5.648 |  |  |  |  |  |  |  |
| 20^th^ | I |  |  | + | + | 1679.261 | 6.126 |  |  |  |  |  |  |  |
| 21^st^ | II | + | + |  | + | 1679.427 | 6.292 |  |  |  |  |  |  |  |
| 22^nd^ | I |  |  | + |  | 1679.568 | 6.433 |  |  |  |  |  |  |  |
| 23^rd^ | IV−8 |  | + |  |  | 1679.761 | 6.626 |  |  |  |  |  |  |  |
| 24^th^ | IV−5 | + |  |  |  | 1681.816 | 8.681 |  |  |  |  |  |  |  |
| 25^th^ | IV−2 | + | + |  |  | 1685.260 | 12.125 |  |  |  |  |  |  |  |

Note: FL, fiber length; FA, fiber area; MOE, modulus of elasticity; MOR, modulus of rupture; CS, compressive strength parallel to grain; TM, tensile Young’s modulus; TS, tensile strength; *Site*_0,_ random effect of site in slope; *Site*_1,_ random effect of site in intercept; *Culm*_0,_ random effect of individual culm in slope; *Culm*_1,_ random effect of individual culm in intercept; AIC, Akaike information criterion; ΔAIC, change in AIC compared to the model with minimum AIC ( the1st place in ‘Model ranking’). Some derived models failed to converge in anatomical and mechanical properties, only the converged models are presented in the table. The symbol of ‘+’ indicates the parameter included in the model. The ΔAIC ≤ 2 indicates no significant difference between models and a simpler model with fewer parameters is preferred. Bold value represents the best model.

**Table S3.** Comparison of AIC values in developed linear and nonlinear mixed-effects models for longitudinal variation of anatomical characteristics and mechanical properties in *B. maculata*

|  | Model Eq. |  | | Random effect | | | | AIC | ΔAIC |  | Model Eq. | Random effect | | | | AIC | ΔAIC |
| --- | --- | --- | --- | --- | --- | --- | --- | --- | --- | --- | --- | --- | --- | --- | --- | --- | --- |
|  |  |  | | *Site*_0_ | *Culm*_0_ | *Site*_1_ | *Culm*_1_ |  |  |  |  | *Site*_0_ | *Culm*_0_ | *Site*_1_ | *Culm*_1_ |  |  |
|  |  | | FL | | | | | | |  | FA | | | | | | |
| 1^st^ | **III** |  | | **+** | **+** |  |  | **27.379** | **0** |  | **IV** |  | **+** |  | **+** | **1049.982** | **0** |
| 2^nd^ | II |  | | + | + |  |  | 28.197 | 0.818 |  | IV |  |  | + | + | 1050.652 | 0.67 |
| 3^rd^ | II |  | | + | + | + |  | 28.649 | 1.27 |  | IV |  |  | + |  | 1052.871 | 2.889 |
| 4^th^ | II |  | | + | + |  | + | 30.187 | 2.808 |  | IV |  |  |  | + | 1054.376 | 4.394 |
| 5^th^ | II |  | | + | + | + | + | 30.649 | 3.27 |  | I |  |  | + | + | 1054.969 | 4.987 |
| 6^th^ | III |  | | + | + | + | + | 31.099 | 3.72 |  | III |  |  | + | + | 1055.094 | 5.112 |
| 7^th^ | II |  | |  | + | + |  | 34.309 | 6.93 |  | III |  | + |  | + | 1055.754 | 5.772 |
| 8^th^ | II |  | | + |  |  | + | 35.306 | 7.927 |  | I |  |  | + |  | 1055.908 | 5.926 |
| 9^th^ | II |  | | + |  | + | + | 36.658 | 9.279 |  | II |  |  | + | + | 1056.679 | 6.697 |
| 10^th^ | III |  | | + |  |  |  | 38.623 | 11.244 |  | II |  |  | + |  | 1057.675 | 7.693 |
| 11^th^ | II |  | | + |  |  |  | 40.782 | 13.403 |  | II |  | + | + |  | 1058.276 | 8.294 |
| 12^th^ | III |  | | + |  | + |  | 42.066 | 14.687 |  | II |  | + | + | + | 1058.679 | 8.697 |
| 13^th^ | II |  | | + |  | + |  | 42.141 | 14.762 |  | I |  |  |  | + | 1058.817 | 8.835 |
| 14^th^ | II |  | |  | + |  |  | 44.04 | 16.661 |  | III |  |  |  | + | 1058.818 | 8.836 |
| 15^th^ | III |  | |  | + |  |  | 44.041 | 16.662 |  | II |  |  |  | + | 1060.527 | 10.545 |
| 16^th^ | III |  | |  | + |  | + | 44.569 | 17.19 |  | IV |  | + |  |  | 1061.215 | 11.233 |
| 17^th^ | II |  | |  |  | + | + | 44.704 | 17.325 |  | III | + |  |  |  | 1061.762 | 11.78 |
| 18^th^ | II |  | |  | + |  | + | 45.124 | 17.745 |  | II | + |  |  | + | 1062.179 | 12.197 |
| 19^th^ | III |  | |  |  | + | + | 45.283 | 17.904 |  | II | + | + |  |  | 1062.92 | 12.938 |
| 20^th^ | IV |  | |  | + |  |  | 46.326 | 18.947 |  | III |  | + |  |  | 1064.23 | 14.248 |
| 21^st^ | II |  | |  |  | + |  | 48.101 | 20.722 |  | II | + |  |  |  | 1064.579 | 14.597 |
| 22^nd^ | III |  | |  |  | + |  | 48.158 | 20.779 |  | II | + | + |  |  | 1065.731 | 15.749 |
| 23^rd^ | IV |  | |  |  | + | + | 54.459 | 27.08 |  | II |  | + |  |  | 1066.517 | 16.535 |
| 24^th^ | II |  | |  |  |  | + | 61.532 | 34.153 |  |  |  |  |  |  |  |  |
| 25^th^ | III |  | |  |  |  | + | 61.987 | 34.608 |  |  |  |  |  |  |  |  |
| 26^th^ | IV |  | |  |  |  | + | 71.163 | 43.784 |  |  |  |  |  |  |  |  |
| 27^th^ | I |  | |  |  | + |  | 176.881 | 149.502 |  |  |  |  |  |  |  |  |
| 28^th^ | I |  | |  |  |  | + | 191.767 | 164.388 |  |  |  |  |  |  |  |  |
|  |  | | MOE | | | | | | |  | MOR | | | | | | |
| 1^st^ | **IV** |  | |  |  | **+** | **+** | **753.316** | **0** |  | IV |  |  | + | + | 1414.367 | 0 |
| 2^nd^ | IV |  | |  |  |  | + | 757.000 | 3.684 |  | **IV** |  | **+** |  |  | **1415.260** | **0.893** |
| 3^rd^ | IV |  | |  | + |  | + | 766.299 | 12.983 |  | III |  |  | + | + | 1423.324 | 8.957 |
| 4^th^ | IV |  | |  |  | + |  | 771.931 | 18.615 |  | III |  | + |  | + | 1425.314 | 10.947 |
| 5^th^ | I |  | |  |  | + | + | 777.893 | 24.577 |  | II |  |  | + | + | 1431.236 | 16.869 |
| 6^th^ | III |  | | + | + |  |  | 779.235 | 25.919 |  | II |  |  |  | + | 1431.339 | 16.972 |
| 7^th^ | II |  | |  |  | + | + | 779.404 | 26.088 |  | II |  | + | + | + | 1432.219 | 17.852 |
| 8^th^ | II |  | |  | + | + |  | 779.648 | 26.332 |  | II |  | + |  | + | 1432.879 | 18.512 |
| 9^th^ | II |  | |  | + | + | + | 780.704 | 27.388 |  | I |  |  | + | + | 1437.494 | 23.127 |
| 10^th^ | II |  | | + |  | + | + | 781.307 | 27.991 |  | I |  |  |  | + | 1437.597 | 23.230 |
| 11^th^ | II |  | | + | + | + |  | 781.648 | 28.332 |  | III |  | + |  |  | 1444.967 | 30.600 |
| 12^th^ | II |  | | + | + | + | + | 782.663 | 29.347 |  | III | + | + |  |  | 1445.726 | 31.359 |
| 13^th^ | I |  | |  |  |  | + | 782.679 | 29.363 |  | II |  | + | + |  | 1455.542 | 41.175 |
| 14^th^ | II |  | |  |  |  | + | 784.189 | 30.873 |  | II |  | + |  |  | 1466.798 | 52.431 |
| 15^th^ | II |  | | + |  |  | + | 785.304 | 31.988 |  | II | + | + |  |  | 1467.538 | 53.171 |
| 16^th^ | I |  | |  |  | + |  | 786.992 | 33.676 |  | IV |  |  | + |  | 1501.583 | 87.216 |
| 17^th^ | II |  | |  |  | + |  | 789.087 | 35.771 |  | III | + |  |  |  | 1506.774 | 92.407 |
| 18^th^ | II |  | | + | + |  |  | 790.169 | 36.853 |  | I |  |  | + |  | 1509.348 | 94.981 |
| 19^th^ | II |  | |  | + |  |  | 792.106 | 38.790 |  | II | + |  |  |  | 1512.932 | 98.565 |
| 20^th^ | II |  | | + |  |  |  | 796.967 | 43.651 |  |  |  |  |  |  |  |  |

Table S3. Continued

| Model rank | Model Eq. | Random effect | | | | AIC | ΔAIC | Model Eq. | Random effect | | | | AIC | ΔAIC |
| --- | --- | --- | --- | --- | --- | --- | --- | --- | --- | --- | --- | --- | --- | --- |
|  |  | *Site*_0_ | *Culm*_0_ | *Site*_1_ | *Culm*_1_ |  |  |  | *Site*_0_ | *Culm*_0_ | *Site*_1_ | *Culm*_1_ |  |  |
|  | CS | | | | | | | TM | | | | | | |
| 1^st^ | III | + | + | + | + | 1146.987 | 0 | **I** |  |  |  | **+** | **1007.136** | **0** |
| 2^nd^ | **III** |  | **+** |  | **+** | **1146.987** | **0** | III |  | + |  | + | 1007.570 | 0.434 |
| 3^rd^ | III |  |  |  | + | 1151.293 | 4.306 | III |  |  |  | + | 1007.756 | 0.62 |
| 4^th^ | IV |  |  |  | + | 1153.791 | 6.804 | I |  |  | + | + | 1008.734 | 1.598 |
| 5^th^ | IV |  |  | + | + | 1155.576 | 8.589 | III |  | + |  |  | 1009.028 | 1.892 |
| 6^th^ | II |  |  |  | + | 1159.102 | 12.115 | III |  |  | + | + | 1009.477 | 2.341 |
| 7^th^ | II |  |  | + | + | 1160.763 | 13.776 | II |  |  |  | + | 1010.499 | 3.363 |
| 8^th^ | II | + |  | + | + | 1162.430 | 15.443 | I |  |  | + |  | 1010.974 | 3.838 |
| 9^th^ | IV |  | + |  |  | 1169.299 | 22.312 | III | + | + |  |  | 1011.028 | 3.892 |
| 10^th^ | I |  |  |  | + | 1172.589 | 25.602 | III |  |  | + |  | 1011.573 | 4.437 |
| 11^th^ | I |  |  | + | + | 1174.250 | 27.263 | II |  |  | + | + | 1012.097 | 4.961 |
| 12^th^ | III |  | + |  |  | 1190.549 | 43.562 | II |  | + |  |  | 1012.540 | 5.404 |
| 13^th^ | II |  | + | + |  | 1199.279 | 52.292 | III | + |  |  |  | 1012.849 | 5.713 |
| 14^th^ | II |  | + |  |  | 1205.937 | 58.950 | II |  | + | + |  | 1013.051 | 5.915 |
| 15^th^ | III |  |  | + |  | 1222.536 | 75.549 | IV |  |  |  | + | 1013.369 | 6.233 |
| 16^th^ | III | + |  |  |  | 1226.734 | 79.747 | II |  |  | + |  | 1014.215 | 7.079 |
| 17^th^ | IV |  |  | + |  | 1226.904 | 79.917 | IV |  |  | + | + | 1015.091 | 7.955 |
| 18^th^ | II |  |  | + |  | 1227.276 | 80.289 | IV |  |  | + |  | 1017.314 | 10.178 |
| 19^th^ | I |  |  | + |  | 1232.171 | 85.184 |  |  |  |  |  |  |  |
| 20^th^ | II | + |  |  |  | 1232.275 | 85.288 |  |  |  |  |  |  |  |
|  | TS | | | | | | |  |  |  |  |  |  |  |
| 1^st^ | **III** |  |  |  | **+** | **1694.419** | **0** |  |  |  |  |  |  |  |
| 2^nd^ | III |  |  | + | + | 1694.942 | 0.523 |  |  |  |  |  |  |  |
| 3^rd^ | III |  | + |  | + | 1695.724 | 1.305 |  |  |  |  |  |  |  |
| 4^th^ | III |  | + |  |  | 1696.341 | 1.922 |  |  |  |  |  |  |  |
| 5^th^ | III |  |  | + |  | 1697.471 | 3.052 |  |  |  |  |  |  |  |
| 6^th^ | II |  |  |  | + | 1697.530 | 3.111 |  |  |  |  |  |  |  |
| 7^th^ | III | + | + |  |  | 1697.637 | 3.218 |  |  |  |  |  |  |  |
| 8^th^ | II |  |  | + | + | 1697.929 | 3.510 |  |  |  |  |  |  |  |
| 9^th^ | IV |  |  | + | + | 1698.259 | 3.840 |  |  |  |  |  |  |  |
| 10^th^ | II | + |  |  | + | 1699.140 | 4.721 |  |  |  |  |  |  |  |
| 11^th^ | III | + |  |  |  | 1699.271 | 4.852 |  |  |  |  |  |  |  |
| 12^th^ | II |  | + | + |  | 1699.337 | 4.918 |  |  |  |  |  |  |  |
| 13^th^ | III | + |  | + |  | 1699.635 | 5.216 |  |  |  |  |  |  |  |
| 14^th^ | II | + |  | + | + | 1699.926 | 5.507 |  |  |  |  |  |  |  |
| 15^th^ | II |  |  | + |  | 1700.430 | 6.011 |  |  |  |  |  |  |  |
| 16^th^ | I |  |  |  | + | 1700.525 | 6.106 |  |  |  |  |  |  |  |
| 17^th^ | II |  | + |  |  | 1700.539 | 6.120 |  |  |  |  |  |  |  |
| 18^th^ | IV |  |  | + |  | 1700.627 | 6.208 |  |  |  |  |  |  |  |
| 19^th^ | I |  |  | + | + | 1700.924 | 6.505 |  |  |  |  |  |  |  |
| 20^th^ | II | + | + |  |  | 1701.734 | 7.315 |  |  |  |  |  |  |  |
| 21^st^ | IV |  | + |  | + | 1701.878 | 7.459 |  |  |  |  |  |  |  |
| 22^nd^ | II | + |  | + |  | 1702.430 | 8.011 |  |  |  |  |  |  |  |
| 23^rd^ | II | + |  |  |  | 1702.483 | 8.064 |  |  |  |  |  |  |  |
| 24^th^ | I |  |  | + |  | 1703.262 | 8.843 |  |  |  |  |  |  |  |

Note: FL, fiber length; FA, fiber area; MOE, modulus of elasticity; MOR, modulus of rupture; CS, compressive strength parallel to grain; TM, tensile Young’s modulus; TS, tensile strength; *Site*_0,_ random effect of site in slope; *Site*_1,_ random effect of site in intercept; *Culm*_0,_ random effect of individual culm in slope; *Culm*_1,_ random effect of individual culm in intercept; AIC, Akaike information criterion; ΔAIC, change in AIC compared to the model with minimum AIC (the 1st place in ‘Model ranking’). Some derived models failed to converge in anatomical and mechanical properties, only the converged models are presented in the table. The symbol of ‘+’ indicates the parameter included in the model. The ΔAIC ≤ 2 indicates no significant difference between models and a simpler model with fewer parameters is preferred. Bold value represents the best model.

**Table S4.** Comparison of AIC values in developed linear and nonlinear mixed-effects models for longitudinal variation of anatomical characteristics and mechanical properties in *G.atter*

| Model rank | Model Eq. | Random effect | | | | AIC | ΔAIC | Model Eq. | Random effect | | | | AIC | ΔAIC |
| --- | --- | --- | --- | --- | --- | --- | --- | --- | --- | --- | --- | --- | --- | --- |
|  |  | *Site*_0_ | *Culm*_0_ | *Site*_1_ | *Culm*_1_ |  |  |  | *Site*_0_ | *Culm*_0_ | *Site*_1_ | *Culm*_1_ |  |  |
|  | FL | | | | | | | FA | | | | | | |
| 1^st^ | II |  | + | + |  | -29.872 | 0 | **IV** |  |  | **+** | **+** | **1023.220** | **0** |
| 2^nd^ | **II** |  | **+** |  |  | **-28.712** | **1.160** | I |  |  | + | + | 1033.978 | 10.758 |
| 3^rd^ | II | + | + |  |  | -27.115 | 2.757 | III |  |  | + | + | 1034.386 | 11.166 |
| 4^th^ | II |  | + |  | + | -27.034 | 2.838 | II | + |  | + | + | 1036.348 | 13.128 |
| 5^th^ | III |  | + |  |  | -26.674 | 3.198 | II |  |  | + | + | 1037.485 | 14.265 |
| 6^th^ | III |  | + |  | + | -25.295 | 4.577 | II | + | + | + | + | 1038.103 | 14.883 |
| 7^th^ | II | + | + |  | + | -25.229 | 4.643 | II |  | + | + | + | 1038.949 | 15.729 |
| 8^th^ | III | + | + |  |  | -25.208 | 4.664 | II |  | + | + |  | 1039.334 | 16.114 |
| 9^th^ | IV |  | + |  |  | -22.258 | 7.614 | II | + | + | + |  | 1039.647 | 16.427 |
| 10^th^ | III |  |  |  | + | -21.649 | 8.223 | IV |  | + |  | + | 1042.236 | 19.016 |
| 11^th^ | II |  |  |  | + | -21.278 | 8.594 | IV |  |  | + |  | 1043.225 | 20.005 |
| 12^th^ | III |  |  | + | + | -21.200 | 8.672 | I |  |  | + |  | 1048.669 | 25.449 |
| 13^th^ | II |  |  | + | + | -20.953 | 8.919 | III |  |  | + |  | 1048.841 | 25.621 |
| 14^th^ | IV |  | + |  | + | -19.659 | 10.213 | II |  |  | + |  | 1051.851 | 28.631 |
| 15^th^ | II | + |  |  | + | -19.457 | 10.415 | I |  |  |  | + | 1052.129 | 28.909 |
| 16^th^ | III |  |  | + |  | -15.188 | 14.684 | III |  |  |  | + | 1052.413 | 29.193 |
| 17^th^ | II |  |  | + |  | -14.434 | 15.438 | II |  |  |  | + | 1055.637 | 32.417 |
| 18^th^ | III | + |  |  |  | -13.525 | 16.347 | III | + | + |  |  | 1065.276 | 42.056 |
| 19^th^ | II | + |  |  |  | -12.165 | 17.707 | III | + |  |  |  | 1070.713 | 47.493 |
| 20^th^ | IV |  |  |  | + | -11.123 | 18.749 | II | + | + |  |  | 1075.268 | 52.048 |
| 21^st^ | IV |  |  | + | + | -10.674 | 19.198 | III |  | + |  |  | 1078.252 | 55.032 |
| 22^nd^ | IV |  |  | + |  | -4.148 | 25.724 | II | + |  |  |  | 1079.046 | 55.826 |
| 23^rd^ | I |  |  | + |  | 111.693 | 141.565 | II |  | + |  |  | 1086.811 | 63.591 |
|  | MOE | | | | | | | MOR | | | | | | |
| 1^st^ | **IV** |  |  | **+** | **+** | **698.903** | **0** | **III** |  |  | **+** | **+** | **1363.056** | **0** |
| 2^nd^ | III | + | + |  |  | 704.947 | 6.044 | II |  | + | + | + | 1366.68 | 3.624 |
| 3^rd^ | IV |  |  |  | + | 709.056 | 10.153 | IV |  |  | + | + | 1369.88 | 6.824 |
| 4^th^ | III |  |  | + | + | 709.141 | 10.238 | II |  | + | + |  | 1370.982 | 7.926 |
| 5^th^ | III |  | + |  | + | 715.389 | 16.486 | II |  |  | + | + | 1371.874 | 8.818 |
| 6^th^ | III |  |  |  | + | 719.294 | 20.391 | III |  | + |  | + | 1375.141 | 12.085 |
| 7^th^ | II | + |  |  | + | 720.229 | 21.326 | III |  |  |  | + | 1375.396 | 12.34 |
| 8^th^ | II | + | + |  | + | 720.353 | 21.450 | II |  | + |  | + | 1383.717 | 20.661 |
| 9^th^ | II | + | + | + | + | 721.425 | 22.522 | II |  |  |  | + | 1384.338 | 21.282 |
| 10^th^ | II |  | + | + | + | 721.647 | 22.744 | II | + | + |  | + | 1385.711 | 22.655 |
| 11^th^ | II | + |  | + | + | 721.659 | 22.756 | II | + |  |  | + | 1386.256 | 23.2 |
| 12^th^ | II |  | + | + |  | 722.140 | 23.237 | III |  | + |  |  | 1393.333 | 30.277 |
| 13^th^ | II | + | + |  |  | 722.249 | 23.346 | II | + | + |  |  | 1404.186 | 41.13 |
| 14^th^ | II | + | + | + |  | 722.670 | 23.767 | II |  | + |  |  | 1413.308 | 50.252 |
| 15^th^ | II |  |  | + | + | 722.796 | 23.893 | III |  |  | + |  | 1423.875 | 60.819 |
| 16^th^ | II |  | + |  | + | 729.124 | 30.221 | II |  |  | + |  | 1429.287 | 66.231 |
| 17^th^ | IV |  |  | + |  | 731.885 | 32.982 | III | + |  |  |  | 1435.973 | 72.917 |
| 18^th^ | II |  | + |  |  | 732.932 | 34.029 | II | + |  |  |  | 1445.635 | 82.579 |
| 19^th^ | II |  |  |  | + | 733.074 | 34.171 | I |  |  | + | + | 1472.153 | 109.097 |
| 20^th^ | III |  |  | + |  | 734.514 | 35.611 | I |  |  |  | + | 1484.617 | 121.561 |
| 21^st^ | I |  |  | + | + | 738.279 | 39.376 | I |  |  | + |  | 1486.915 | 123.859 |
| 22^nd^ | II | + |  |  |  | 743.246 | 44.343 |  |  |  |  |  |  |  |
| 23^rd^ | II |  |  | + |  | 744.219 | 45.316 |  |  |  |  |  |  |  |
| 24^th^ | II | + |  | + |  | 744.511 | 45.608 |  |  |  |  |  |  |  |
| 25^th^ | I |  |  |  | + | 748.557 | 49.654 |  |  |  |  |  |  |  |
| 26^th^ | I |  |  | + |  | 753.358 | 54.455 |  |  |  |  |  |  |  |

Table S4. Continued

| Model rank | Model Eq. | Random effect | | | | AIC | ΔAIC | Model Eq. | Random effect | | | | AIC | ΔAIC |
| --- | --- | --- | --- | --- | --- | --- | --- | --- | --- | --- | --- | --- | --- | --- |
|  |  | *Site*_0_ | *Culm*_0_ | *Site*_1_ | *Culm*_1_ |  |  |  | *Site*_0_ | *Culm*_0_ | *Site*_1_ | *Culm*_1_ |  |  |
|  | CS | | | | | | | TM | | | | | | |
| 1^st^ | III | + | + | + | + | 1095.595 | 0 | **I** |  |  | **+** | **+** | **1004.965** | **0** |
| 2^nd^ | **III** |  |  | **+** | **+** | **1096.866** | **1.271** | III |  |  | + | + | 1005.250 | 0.285 |
| 3^rd^ | III |  | + |  | + | 1105.618 | 10.023 | II |  | + | + |  | 1007.266 | 2.301 |
| 4^th^ | III |  |  |  | + | 1107.620 | 12.025 | III |  |  | + |  | 1007.347 | 2.382 |
| 5^th^ | II | + |  | + | + | 1107.982 | 12.387 | II |  |  | + | + | 1008.455 | 3.490 |
| 6^th^ | II |  |  | + | + | 1109.380 | 13.785 | II | + | + | + |  | 1009.266 | 4.301 |
| 7^th^ | IV |  |  |  | + | 1109.693 | 14.098 | IV |  |  | + | + | 1010.144 | 5.179 |
| 8^th^ | II |  | + | + | + | 1111.338 | 15.743 | III | + | + |  |  | 1010.245 | 5.280 |
| 9^th^ | II |  |  |  | + | 1120.258 | 24.663 | II | + |  | + | + | 1010.455 | 5.490 |
| 10^th^ | II | + |  |  | + | 1121.359 | 25.764 | II |  |  | + |  | 1010.495 | 5.530 |
| 11^th^ | II |  | + | + |  | 1145.433 | 49.838 | III | + |  |  |  | 1012.629 | 7.664 |
| 12^th^ | II | + | + | + |  | 1147.369 | 51.774 | III |  |  |  | + | 1014.747 | 9.782 |
| 13^th^ | III | + | + |  |  | 1157.610 | 62.015 | II | + |  |  | + | 1015.949 | 10.984 |
| 14^th^ | I |  |  | + | + | 1161.779 | 66.184 | II | + | + |  |  | 1016.735 | 11.770 |
| 15^th^ | I |  |  |  | + | 1172.656 | 77.061 | III |  | + |  |  | 1017.048 | 12.083 |
| 16^th^ | II | + | + |  |  | 1183.550 | 87.955 | II |  |  |  | + | 1018.075 | 13.110 |
| 17^th^ | III |  |  | + |  | 1185.751 | 90.156 | III |  | + |  | + | 1018.449 | 13.484 |
| 18^th^ | IV |  |  | + |  | 1190.412 | 94.817 | II | + |  |  |  | 1018.571 | 13.606 |
| 19^th^ | II |  |  | + |  | 1191.818 | 96.223 | IV |  |  |  | + | 1019.640 | 14.675 |
| 20^th^ | II |  | + |  |  | 1192.903 | 97.308 | II |  | + |  |  | 1022.479 | 17.514 |
| 21^st^ | III | + |  |  |  | 1200.696 | 105.101 | I |  |  |  | + | 1172.656 | 167.691 |
| 22^nd^ | II | + |  |  |  | 1211.841 | 116.246 | I |  |  | + |  | 1212.348 | 207.383 |
| 23^rd^ | I |  |  | + |  | 1212.348 | 116.753 |  |  |  |  |  |  |  |
|  | TS | | | | | | |  |  |  |  |  |  |  |
| 1^st^ | **III** |  |  | **+** | **+** | **1671.255** | **0** |  |  |  |  |  |  |  |
| 2^nd^ | II |  | + | + |  | 1674.382 | 3.127 |  |  |  |  |  |  |  |
| 3^rd^ | II |  |  | + |  | 1674.398 | 3.143 |  |  |  |  |  |  |  |
| 4^th^ | IV |  |  | + | + | 1674.646 | 3.391 |  |  |  |  |  |  |  |
| 5^th^ | II |  |  | + | + | 1674.954 | 3.699 |  |  |  |  |  |  |  |
| 6^th^ | III | + | + |  |  | 1675.394 | 4.139 |  |  |  |  |  |  |  |
| 7^th^ | II |  | + | + | + | 1676.381 | 5.126 |  |  |  |  |  |  |  |
| 8^th^ | III |  |  |  | + | 1678.713 | 7.458 |  |  |  |  |  |  |  |
| 9^th^ | III |  | + |  |  | 1679.866 | 8.611 |  |  |  |  |  |  |  |
| 10^th^ | II | + |  |  |  | 1680.493 | 9.238 |  |  |  |  |  |  |  |
| 11^th^ | II | + |  |  | + | 1680.844 | 9.589 |  |  |  |  |  |  |  |
| 12^th^ | I |  |  | + |  | 1681.688 | 10.433 |  |  |  |  |  |  |  |
| 13^th^ | IV |  |  |  | + | 1682.105 | 10.850 |  |  |  |  |  |  |  |
| 14^th^ | II |  |  |  | + | 1682.537 | 11.282 |  |  |  |  |  |  |  |
| 15^th^ | I |  |  | + | + | 1682.677 | 11.422 |  |  |  |  |  |  |  |
| 16^th^ | II | + | + |  | + | 1682.705 | 11.450 |  |  |  |  |  |  |  |
| 17^th^ | II |  | + |  | + | 1684.525 | 13.270 |  |  |  |  |  |  |  |
| 18^th^ | II |  | + |  |  | 1684.845 | 13.590 |  |  |  |  |  |  |  |
| 19^th^ | II | + | + |  |  | 1689.885 | 18.630 |  |  |  |  |  |  |  |
| 20^th^ | I |  |  |  | + | 1690.260 | 19.005 |  |  |  |  |  |  |  |

Note: FL, fiber length; FA, fiber area; MOE, modulus of elasticity; MOR, modulus of rupture; CS, compressive strength parallel to grain; TM, tensile Young’s modulus; TS, tensile strength; *Site*_0,_ random effect of site in slope; *Site*_1,_ random effect of site in intercept; *Culm*_0,_ random effect of individual culm in slope; *Culm*_1,_ random effect of individual culm in intercept; AIC, Akaike information criterion; ΔAIC, change in AIC compared to the model with minimum AIC (the 1st place in ‘Model ranking’). Some derived models failed to converge in anatomical and mechanical properties, only the converged models are presented in the table. The symbol of ‘+’ indicates the parameter included in the model. The ΔAIC ≤ 2 indicates no significant difference between models and a simpler model with fewer parameters is preferred. Bold value represents the best model.
